# Ultrasonic Vibration Assisted Electro-Discharge Machining (EDM)—An Overview

**DOI:** 10.3390/ma12030522

**Published:** 2019-02-10

**Authors:** Nurbol Sabyrov, M. P. Jahan, Azat Bilal, Asma Perveen

**Affiliations:** 1 Department of Mechanical & Aerospace Engineering, Nazarbayev University, Astana 010000, Kazakhstan; nurbol.sabyrov@nu.edu.kz (N.S.); azat.bilal@nu.edu.kz (A.B.); 2 Department of Mechanical Engineering, Miami University, Oxford, OH 45056, USA; jahanmp@miamioh.edu

**Keywords:** EDM, ultrasonic vibration, material removal rate, surface roughness, hard to cut materials

## Abstract

Many of the industrial processes, including material removal operation for shape generation on the surface of material, exploit the assistance of ultrasonic vibrations. This trend of using ultrasonic vibration in order to improve the process performance is becoming more and more prominent recently. One of the significant applications of this ultrasonic vibration is in the industrial processes such as Electro-discharge machining (EDM), where ultrasonic vibration (UV) is inserted as a medium for enhancing the process performance. Mostly ultrasonic vibration is applied along with the EDM process to increase the efficiency of the process through debris cleansing from the sparking gap. There have been significant changes in ultrasonic assisted technology during the past years. Due to its inherent advantages, ultrasonic assistance infiltrated in different areas of EDM, such as wire cut EDM, micro EDM and die sinking EDM. This article presents an overview of ultrasonic vibration applications in electric discharge machining. This review provides information about modes of UV application, impacts on parameters of performance, optimization and process designing on difficult-to-cut materials. On the bases of available research works on ultrasonic vibration assisted EDM, current challenges and future research direction to improve the process capabilities are identified. Literature suggested improved material removal rate (MRR), increased surface roughness (SR) and tool wear ratio (TWR) due to the application of ultrasonic vibration assisted EDM. However, tool wear and surface roughness can be lessened with the addition of carbon nanofiber along with ultrasonic vibration. Moreover, the application of ultrasonic vibration to both tool and workpiece results in higher MRR compared to its application to single electrode.

## 1. Introduction

Electric discharge machining (EDM) is a non-conventional process based on electro-thermal energy and is used for machining of diverse materials with complex geometrical shapes as well as precise dimensional cuttings. EDM process is especially well recognized for manufacturing of hard-to-machine materials. By overcoming technical difficulties associated with the conventional machining, EDM technology finds its appropriate applications not only in the aerospace & automotive industries but also in the surgical and medical implant creation [1,2]. In addition, mould and die fabrication [3], burr free micro hole or slot fabrication [4] also exploit the EDM technology due to the hardness issue of the materials and larger cutting force involved in the conventional machining [5]. However, EDM process also suffers in terms of performance. Quite a lot of investigations have been pursued to improve the machining process of EDM, especially focusing on the following aspects: such as increasing material removal rate, enhancing surface quality and reducing tool wear rate [1,6].

During the EDM process, when voltage between two electrodes is sufficiently high enough to break the dielectric strength, pulse discharge current generated between tool and workpiece aids in materials removal. Melting and vaporization of specimen material are caused by the dielectric liquid breakdown. The removed debris materials gather in the machining gap which eventually decreases the resistance in this gap [7]. Consequently, it becomes the reason behind the occurrences of abnormal discharges in the short circuit. As a result, significant increase in tool wear become obvious and MRR becomes slower [8]. Therefore, quick cleansing of debris from the sparking gap contributes to the enhancement of efficiency and this phenomena plays a crucial role on the process performance [9]. Research on improved process efficiency so far reported different solutions such as different flushing mechanism, various type of dielectric media, magnetic felid assisted EDM, coated electrode and so forth. [10]. Investigation dedicated to the development of flushing methods for cleaning the debris from the spark gap was one of the main research areas for the past years. Implementation of the ultrasonic vibration during EDM process is found to be advantageous and became one of the methods of debris evacuation. Nowadays, in the EDM industry there exists this hybrid machining process, in which low-amplitude vibrations of a certain frequency are applied on one of following parts: dielectric fluid, workpiece or tool for increasing the machining efficiency through flushing strengthening mechanism [11]. The principle of this method is based on the rapid change of sparking gap and producing a high frequency altering pressure change of the dielectric into the spark gap by ultrasonically vibrating the workpiece or electrode. That kind of fluctuating pressure helps in the elimination of molten materials. Moreover, ultrasonic vibration aids in perfect dielectric circulation, thus, removing the accumulated debris particles from the working gap. As a result, high MRR is obtained through effective debris removal and its consequence on discharge effectiveness [12].

The general purpose of the article is to unfold the underpinning knowledge of Ultrasonic vibration assisted EDM process, its performance parameters, modes of applications, process modelling, metallurgical characteristics on the surface and optimization of the process.

## 2. Principle of Processes

### 2.1. Basic Mechanism of EDM/Micro-EDM

**Figure 1:**
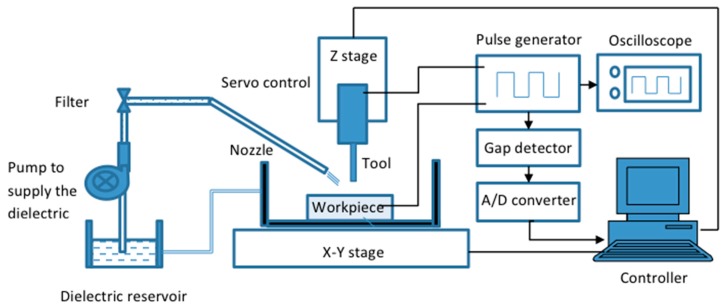
Schematic diagram of the EDM system [15]. (Adapted from [15] with permission—© 2014 Springer.

Electrical discharge machining (EDM) is the method of removing electrically conductive materials in the form of small craters ranging from several to tens of microns using strictly coordinated sparks occurring between a tool electrode and a workpiece in the presence of a dielectric fluid [13]. The mechanism of the micro-EDM process is fundamentally the same as the EDM process with the major differences being tool dimensions, discharge energy supplied and the resolution of the axes movement [14]. The material removal occurs due to the series of sparks in a controlled spark gap, that is, gap between the tool and the electrode, where every single spark removes a tiny amount of material from the workpiece in the form of crater. The series of sparks are allowed for a certain period of time named as pulse duration [µs], which is followed by a pause in the sparking process for certain time duration called pulse interval [µs]. Each discharge cycle consists of pulse duration and pulse interval. The discharge frequency can be controlled by controlling how many cycles could run in a second. The discharge energy for a single pulse can be controlled by the pulse generator based on the machining conditions, that is, roughing, semi-finishing or finishing. A servo control system is engaged to control the rate at which the electrode moves toward the workpiece and to maintain the spark gap, as well as to withdraw the electrode if short-circuiting happens. In order to maintain the fresh dielectric flow in the spark gap and effective flushing out of debris from the machined zone, a dielectric circulation system comprising pump, filter and dielectric reservoir is built in the EDM/micro-EDM system. Figure 1 shows the basic EDM/Micro-EDM system.

**Figure 2:**
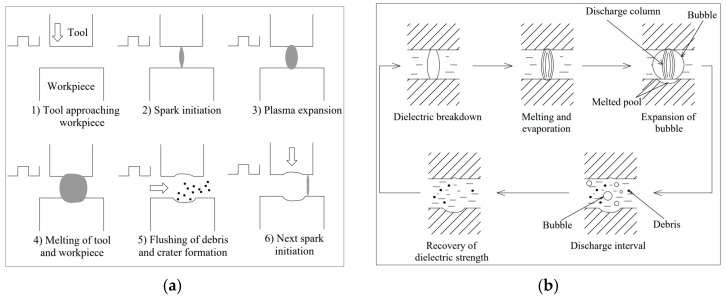
(**a**) Representation of sparking phenomena in EDM [18]; (Adapted from [18] with permission—© 2006 Elsevier). (**b**) Model of EDM gap phenomena [19] in EDM. (Adapted from [19] with permission—© 2005 Elsevier).

The sparking and gap phenomena in EDM and micro-EDM are comprised of three main phases, preparation for the sparking to occur, sparking phase itself and interval phase between two consecutive sparking phases [16]. At the beginning of the machining, the voltage is applied across the spark gap, an electric field is created surrounding the spark gap that consists of an energy column. As the sparking continues, the energy column expands. The energy column’s strength is higher where the spark gap is smaller. The breakdown of the dielectric happens allowing the current to flow through the spark gap. As the current flows, the voltage drops, the energy column collapses. The spark vaporizes the material from both the electrode and the workpiece by melting and evaporation thus resulting in a crater on both workpiece and electrode. The size of craters depends on the polarity of the electrode and workpiece and can be controlled based on the requirements. Material removal energy is reported to be 3% of total energy consumed by machine tool and EDM process efficiency is below 0.01% [17]. After the material is removed, the sparking process ceases and pulse interval takes place. During this time, the removed debris are cleared away from the spark gap by the flushing mechanism, which also brings fresh dielectric into the spark gap and the system gets ready for new sets of sparking. The sparking [18] and gap [19] phenomena during the EDM/micro-EDM process are shown schematically in the Figure 2.

### 2.2. Need for UV Assisted EDM

One of the major challenges of EDM and micro-EDM is the low throughput/MRR. This challenge becomes even more evitable during the micro scale machining of blind features. As the UV assisted EDM combines the effect of ultrasonic cleaning at the narrow spark gap of few microns with the traditional discharging induced material removal, the overall MRR increases. This is mainly due to two reasons. The ultrasonic vibration induces cavitation bubbles into the dielectric liquid at the spark gap, resulting in micro jets, which aids in removal of debris from the machined zone effectively. In addition, due to the instantaneous and continuous removal of debris and circulation of fresh dielectric into the spark gap, the discharge frequency increases, which further increases the MRR.

Another challenge in EDM and micro-EDM is the machining instability due to very small spark gap (in the range of microns), resulting in a long machining time, high electrode wear and surface defects. During machining of deep holes or complex 3D microstructures using die-sinking micro-EDM, removal of debris from the small spark gap becomes challenging. The debris, if not removed instantly and efficiently, creates a bridge of debris particles resulting in a short circuiting between the workpiece and the electrode, thus ceasing the machining operations. In addition, due to the higher concentration of debris particles in the small spark gap, the arcing (continuous sparking) occurs on the debris particles causing the adhesion of debris particles on the machined surface and resulting in surface and sub-surface defects. Ultrasonic vibration assisted EDM has the capability of solving those two major challenges in EDM and micro-EDM, thus enhancing the capability of EDM in deep hole micromachining and complex 3D microstructures. The UV assisted EDM can also be useful in machining of large 3D structures using conventional die-sinking EDM and high aspect ratio and thin slot cutting using wire EDM.

### 2.3. Principle of UV Assisted EDM

**Figure 3:**
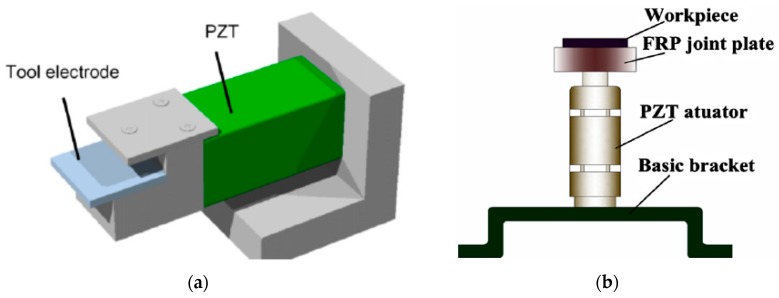

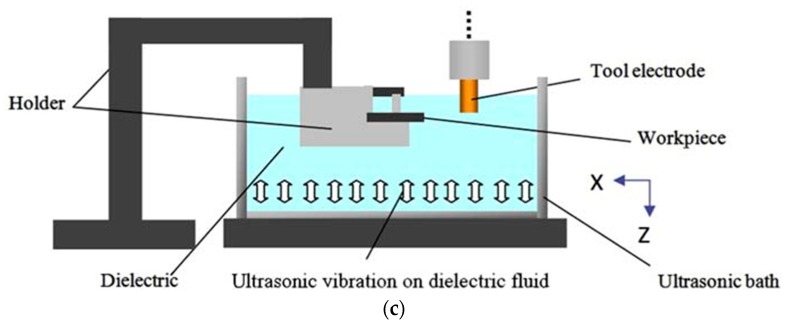
Principle of UV assisted EDM/micro-EDM; (**a**) vibration applied to tool electrode [8]; (Adapted from [8] with permission—© 2008 Elsevier). (**b**) vibration applied to workpiece [20] (Adapted from [20] with permission—© 2008 Elsevier).and (**c**) vibration applied to dielectric [21]. (Adapted from21] with permission—© 2009 Elsevier).

Ultrasonic vibration (UV) assisted EDM/micro-EDM is a hybrid process that combines ultrasonic vibration to the workpiece, electrode or dielectric during the conventional EDM or micro-EDM process. In this process, the ultrasonic vibration (frequency of 20 kHz or above) is introduced during the EDM process to improve the flushing efficiency of the process. Based on the applications and the challenges faced during the EDM/micro-EDM process, the ultrasonic vibration can be applied to the tool electrode [8], workpiece [20] or even dielectric [21]. The process mechanisms of each of the cases are shown in Figure 3 and are discussed in brief in the following sections.

#### 2.3.1. Ultrasonic Vibration Applied to Tool Electrode

In the tool vibration assisted EDM, the ultrasonic vibration is applied to the tool electrode through a high frequency piezoelectric transducer that is connected to the tool electrode through a holder (See Figure 3a). The ultrasonic vibration is applied to the PZT actuator from the pulse generator. The feed direction of the tool electrode can be parallel to the direction of vibration of the PZT or perpendicular to the direction of the vibration of the PZT. For the system where feed direction is parallel to the vibration, an assembly of collar, spring and holding device is used to transmit the vibration from the transducer to the tool electrode. In this method of tool electrode vibration, owing to the suction and ultrasonic vibration, dielectric circulation and debris removal are improved significantly. Tool vibration is comparatively more difficult to apply in micro-EDM, as the diameter of the tool electrode is only several microns; hence, there is a chance of tool deflection.

#### 2.3.2. Ultrasonic Vibration Applied to Workpiece

In this system, the ultrasonic vibration in the form of sinusoidal wave with desired frequency are generated by the pulse generator and transmitted to the piezoelectric transducer (PZT). The PZT actuator is connected to the workpiece through a joint plate, which transfers the ultrasonic vibration to the workpiece, as shown in Figure 3b. An online feedback control system is used for monitoring the spark gap and thus maintaining the machining stability [22]. During the workpiece vibration, the forward and backward movement of the workpiece causes the changes into the spark gap and dielectric liquid pressure with time, thus maintaining a sinusoidal relationship of spark gap and pressure difference with time [23].These two phenomena force the debris out of the spark gap and improve the flushing efficiency by continuously supplying the fresh dielectric into the spark gap, thus improving the machining stability and MRR(Material removal rate) significantly.

#### 2.3.3. Ultrasonic Vibration Applied to Dielectric Liquid

The ultrasonic vibration can also be applied to the dielectric fluid to create the turbulence into the small spark gap between the electrode and the workpiece, and effectively flush away the debris from the machined zone (Figure 3c). Several studies have focused on applying ultrasonic vibration during the EDM and micro-EDM process, especially using powder mixed EDM [21]. The application of ultrasonic vibration in powder mixed dielectric serves two functions. First, it can eliminate the need for stirrer and maintain uniform concentration of powder in the dielectric. Second, it can serve the purpose of the cleaning of debris effectively from the spark gap by creating turbulence in the fluid at the narrow gap between the workpiece and the electrode. The ultrasonic vibration to the liquid helps minimizing the accumulation and deposition of powders at the bottom of the EDM tank, thus improving the effectiveness of the process and lessening the surface defects.

### 2.4. UV Assisted EDM in Gas Medium

**Figure 4:**
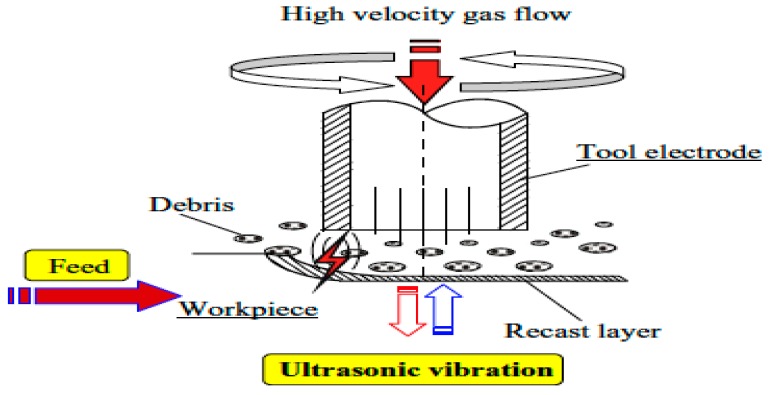
Schematic diagram illustrating the mechanism of ultrasonic vibration assisted EDM in gas [24]. (Adapted from [24] with permission—© 2017 Springer).

The ultrasonic vibration assisted EDM in gas medium is basically a hybrid process that combines the ultrasonic vibration to the workpiece during dry EDM (or EDM with gas as a dielectric). In dry EDM, high pressured gas is used as a dielectric fluid that is supplied mainly through the centre of the tool electrode during the EDM process. In dry EDM, short circuiting happens frequently due to the narrow spark gap of few microns and comparatively higher applied voltage. As a result, in order to enhance the machining stability and MRR, the ultrasonic vibration is applied to the workpiece in addition to tool rotation. Both the tool rotation and ultrasonic vibration helps in reducing the short-circuiting and thus enhancing the machining stability. The ultrasonic vibration further facilitates the removal of molten material and resolidified debris from the workpiece and the narrow spark gap, thus generating the sparking on the fresh surface of the workpiece and improving the MRR. Figure 4 presents the working principle of the ultrasonic vibration assisted EDM in gas medium [24]. Table 1 gives an overview of research summary conducted in UV assisted Gas EDM.

## 3. Modelling of UV Assisted EDM

### 3.1. Modelling of MRR and SR

There have been several studies attempting to model ultrasonic vibration assisted EDM, both in liquid and gas dielectric mediums. One of the early studies on the UV assisted EDM in liquid dielectric by Zhang et al. (2006) [31] developed theoretical models for MRR and surface roughness. They have made a number of assumptions about the EDM and discharging process, such as each pulse is made of one spark, the crater formed in a single spark is of spherical form, ignition delay time is short and constant and each effective pulse has similar shape waveforms.

The analytical equation derived for calculating the MRR is as follows [31]:

(1) $$M=\frac{V_{tot}}{T_{tot}}=\frac{\eta_{1}\int_{0}^{t_{s}}I_{e}\left(t\right)U_{e}\left(t\right)dt}{t_{s}+t_{i}}$$

where, *M* = MRR, *V_tot_* = summation of the volume removed by all single sparks, *T_tot_* = total time duration, η_1_ = proportional constant, *I_e_* = discharge current, *U_e_* = discharge voltage, *t_i_* is the idling time, *t_s_* is the pulse duration.

**Figure 5:**
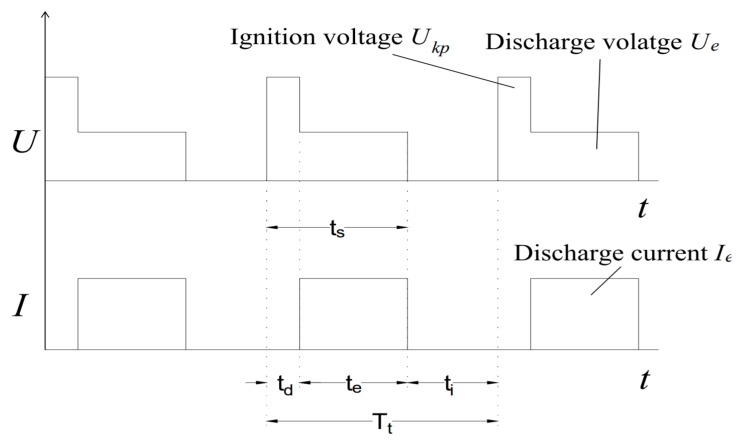
Discharge current and voltage waveforms showing pulse duration, pulse interval and ignition delay [31]. (Adapted from [31] with permission—© 2006 Elsevier).

The equation can be further simplified for the assumption that all the discharge pulses are square, as shown in Figure 5. Thus, the equation for MRR becomes:

(2) $$M=\frac{\eta_{1}I_{e}U_{e}t_{s}}{t_{s}+t_{i}}$$

**Figure 6:**
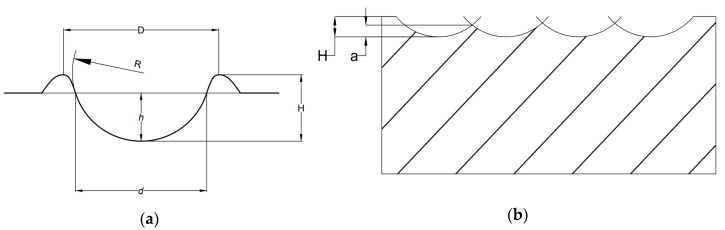
Illustration of the crater geometry and surface roughness due to series of crater formation [31] (Adapted from [31] with permission—© 2006 Elsevier).

In order to model the surface roughness, Zhang et al. (2006) assumed that the crater formed by a single spark is of spherical crest size with radius R and crater depth H, as shown in Figure 6a. By using the geometry to calculate the volume of the craters and equating that with the equation of *V_tot_*, they were able to calculate/estimate the values of average surface roughness *R_a_*. (Figure 6b) The analytical equation for *R_a_* was proposed as follows [31]:

(3) $$R_{a}=\xi_{1}{\left(I_{e}U_{e}t_{s}\right)}^{\frac{1}{3}}$$

where, η_1_ = proportional constant, *I_e_* = current, *U_e_* = discharge voltage, *t_s_* is the pulse duration.

**Figure 7:**
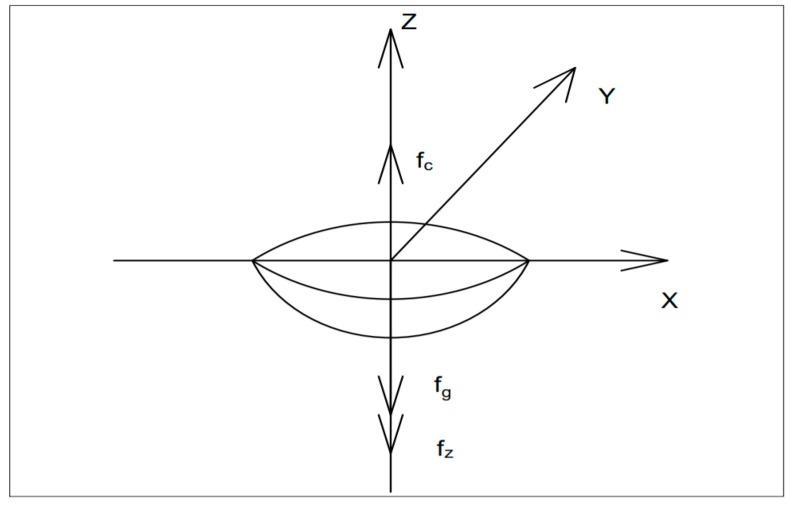
The strength of the molten metal drop model [32]. (Adapted from [32] with permission—© 2009 Elsevier).

The same research group, in a later study led by Xu et al. (2010) further modified the equation of MRR for the UV assisted EDM in gas. Considering the same geometry of the crater, spherical crest, as shown in Figure 7, they found that the MRR can be expressed as follows [32]:

(4) $$MRR=\frac{V}{t_{0}+t_{d}}=\frac{\xi I_{e}U_{e}t_{o}}{t_{0}+t_{d}}+\frac{4\eta a^{3}\pi^{2}T}{(t_{0}+t_{d})3\rho f^{2}}=\frac{3\xi \rho f^{2}I_{e}U_{e}t_{o}+4\eta a^{3}\pi^{2}T}{(t_{0}+t_{d})3\rho f^{2}}$$

where, *MRR* = material removal rate, *V* = summation of the volume of material removed by all single sparks, *t*_o_ is discharge pulse-on time and *t_d_* is pulse-off time, η is a coefficient. *ρ* is Density, *f* is the frequency and *a* is the amplitude of ultrasonic, *T* = total time duration, η_1_ = proportional constant, *I_e_* = current, *U_e_* = discharge voltage, and

And for crater diameter *D* and crater height *H*, the following constant was simplified in the model.

(5) $$\xi =\frac{K\eta }{6.24\pi }$$

(6) $$K=\frac{\pi }{8}{\left(\frac{D}{H}\right)}^{2}$$

### 3.2. Modelling of Recast Layer Thickness

Shabgard et al. (2017) [37] carried out analytical and numerical modelling as well as experimental validation for the recast layer thickness during the ultrasonic vibration assisted EDM. They started from the Gaussian heat flux thermal model and was able to estimate the recast layer thickness based on the heat source, crater dimensions, plasma channel radius, fraction of energy transferred to the workpiece and plasma flushing efficiency.

The numerical value of recast layer thickness (*RLT_Num_*) has been calculated as follows:

(7) $$RLT_{Numerical}=D_{C}-\%PFE\times D_{C}$$

where, *D_c_* = Depth of crater and %*PFE* = plasma flushing efficiency

The plasma flushing efficiency can be obtained as:

(8) $$\%PFE=100\times \frac{V_{C}\left(EXP\right)}{V_{C}\left(FEM\right)}$$

where, *V_C_*(*FEM*) is the theoretical volume of melted material per pulse and *V_C_(EXP)* is the experimental volume of removed material per pulse.

The experimental volume of removed material per pulse (*V_C_*(*EXP*)) is obtained by:

(9) $$V_{C}\left(EXP\right)=\frac{\left(M_{1}-M_{2}\right)}{\left(NN\times \rho \right)}$$

where *M*_1_ and *M*_2_ are the weight of workpiece before and after machining, respectively, *NN* is the number of normal pulses at the end of machining period and *ρ* is the workpiece material density.

The theoretical crater volume obtained by FEM analysis, with the assumption of a circular parabolic geometry for the crater is:

(10) $$V_{C}\left(FEM\right)=\frac{1}{2}\pi D_{C}R_{C}{}^{2}$$

where *D_C_* and *R_C_* are the depth and radius of the crater, considering the crater is of spherical shape as shown in Figure 6.

### 3.3. Modelling of Debris Removal Mechanism

There have been a few studies on the modelling of the debris removal mechanism during UV assisted EDM. Understanding the physics of the process on how the UV assistance promotes the debris removal from the spark gap is of prime importance. Xu et al. (2009) [32] established an analytical modelling that melting liquid drop can easily overcome the inertia force of the crater under the influence of ultrasonic vibration. They have carried out the model for the ultrasonic vibration assisted EDM in the gas medium to show how ultrasonic vibration can assist in the removal of debris, even without the flushing mechanism in place. Figure 7 shows the forces acting on the crater, where f_c_ is inertia force caused by ultrasonic vibration and f_g_ is gravity, which is so small compared to f_c_ that it can be ignored and f_z_ is surface tension of liquid drops.

The developed equation for the maximum inertia force of the molten metal drop is as follows:

(11) $$F_{max}=ma_{max}=my_{max}{}^{\prime \prime }=4m\pi^{2}f^{2}A$$

(12) $$Y\left(t\right)=A\sin\left(\frac{2\pi f}{c}x\right)\sin\left(2\pi ft\right)$$

where, *m* = mass of the molten metal droplet, *a_max_* = *y*″*_max_* = maximum acceleration of the molten drop, where *f* is the frequency of ultrasonic vibration, *A* is the amplitude of ultrasonic vibration, *t* = time.

The maximum inertia force for the crater can be calculated as:

(13) $$F_{max}=\frac{2}{3}\rho H\pi^{3}f^{2}\left[3{\left(\frac{D}{2}\right)}^{2}+H^{2}\right]$$

where *f* is the frequency of ultrasonic vibration, *A* is the amplitude of ultrasonic vibration, *D* is the diameter of the discharge crater and H is the depth of the discharge crater.

And the surface tension of the liquid drops can be obtained by the following formula [38]:

(14) $$\sigma_{a}=\frac{\frac{\frac{100M\rho }{T_{m}}}{C_{1\;}}}{C_{2}}$$

where *M* is a coefficient, *ρ* is the density of the metal, *T_m_* is the melting point of the metal, *C*_1_ is adjusting coefficient of density and *C*_2_ is adjustment coefficient of melting point.

From the above three equations, it was easily determined that melting liquid drops could be easily shot off by the effect of inertia force caused by ultrasonic vibration, thus improving the effectiveness of the ultrasonic vibration assisted EDM.

## 4. Research on Ultrasonic Vibration Assisted EDM

Various types of UV assisted EDM have been reported in the literature. These studies can be classified into three categories, such as, vibration to tool/wire, vibration to workpiece and vibration to dielectric. This section presents the summary of research studies carried out on these three kinds of UV assisted EDM.

### 4.1. Ultrasonic Vibration of Tool and Wire Electrode

**Figure 8:**
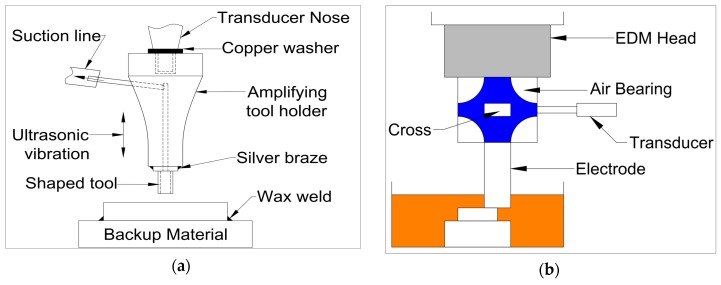
(**a**) Ultrasonic Unit (**b**) Ultrasonic vibrating EDM [40]. (Adapted from [40] with permission—© 1989 Elsevier).

For achieving better performance, tool horn should match the resonant frequency of the transducer (Figure 8a). Nanu et al. [39] carried out finite element study of horn design using ANSYS that included the UV frequency distribution at different part of the horn. This analysis helps in finding the right dimension of horn for achieving longitudinal mode vibration. Synchronization of ultrasonic generator and EDM pulse generator is imperative for best performance of UVA-EDM.

**Figure 9:**
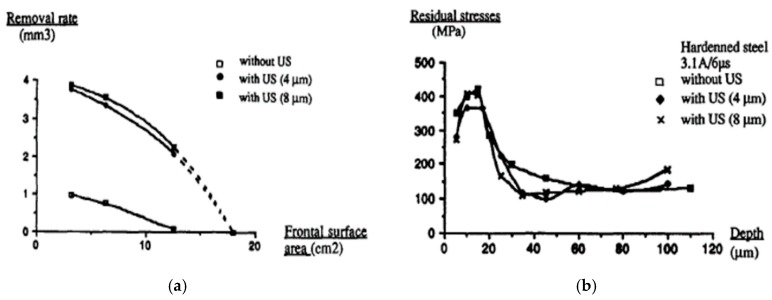
(**a**) Effect to vibration on Removal rate; (**b**) Residual stress [40]. (Adapted from [40] with permission—© 1989 Elsevier).

Figure 8b shows a schematic representation of vibration assisted EDM as demonstrated by Kremer et al. [40]. Their study focused on the MRR, EWR, roughness, hardness, fatigue strength and white layer generation characteristic during UV assisted EDM. The authors reported that the process performance of vibrating tool is better than the non-vibrating tool and the reason might be the better flushing condition achieved by the vibration as well as effective discharging. Although ultrasonic vibration assisted EDM shows improved MRR (Figure 9a) and surface roughness, it also deteriorates the tool wear condition (10–15% increases) due to the porous nature of graphite tool which reacts to the cavitation mechanism of the process. Heat effected zone reported by their study is smaller than EDM and it comes with regular as well as continuous white layer. In addition, tempered and reheated layer appears to be thinner as well. However, both hardness and residual stress are not much affected by ultrasonic vibration assisted EDM (Figure 9b).

**Figure 10:**
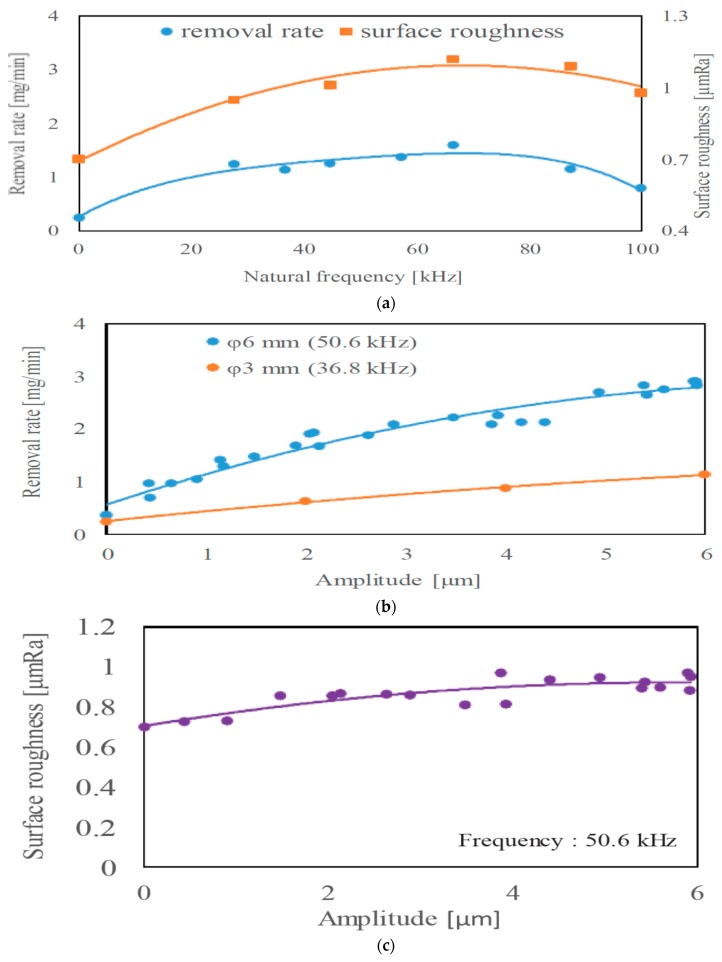
(**a**) Relationship between vibration frequency, removal rate and surface roughness; (**b**) Comparison of removal rate for each electrode; (**c**) Relationship between vibration frequency and surface roughness [41]. (Adapted from [41] with permission—© 2018 Elsevier).

In the research of Hirao et al. [41], the application of ultrasonic vibration on tool electrode resulted in an increase of machining speed even if the amplitude of vibration was 1 µm. Furthermore, the rate of abnormal discharges, which occurred because of the bridging contact between the electrode, accumulated dust and workpiece, was decreased since the tool was forcibly separated from the machined workpiece by this “pumping action.” Such tendency has a positive effect on the surface finish because of an increase in the rate of normal discharges. Comparative analysis of EDM and ultrasonic vibration assisted EDM shows not much difference in generated roughness with the change of ultrasonic vibration frequency, however, MRR seems to be improving until 66.5 kHz and then drops gradually (Figure 10a). In addition, MRR also increases with the increase of the tool diameter as well as the ultrasonic vibration amplitude (Figure 10b). As can be seen in Figure 10c, UV assisted EDM with 6 µm of amplitude increases roughness of 0.2 µm compared to EDM.

**Figure 11:**
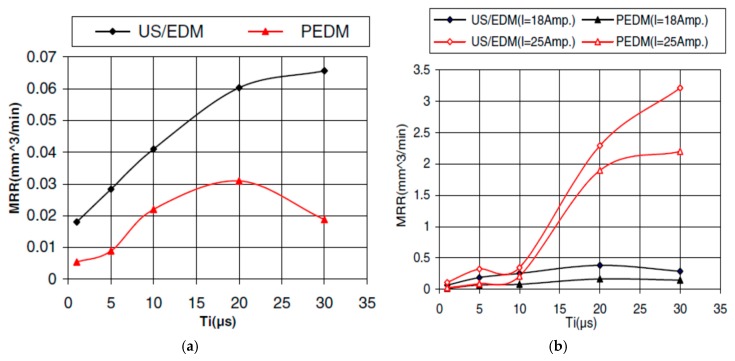
(**a**) Effect of tool vibration on MRR against pulse-on time (Ti); (**b**) pulse-on time (Ti) [42]. (Adapted from [42] with permission—© 2007 Springer).

Shervani-Tabar et al. [42] investigated the dynamics of vapor bubble formation during EDM process and how it is effected by vibration application numerically by solving the boundary integral equation. During conventional EDM process, bubble generated due to electric discharge stays in somewhere middle point between the workpiece and tool with its initial volume. When ultrasonic vibration is applied to EDM, two cases are considered, first one considers discharge occurrence when tool and workpiece are in closer distance, second one considers discharge occurrence at the far distance of tool and workpiece. For second case, during bubble growth and collapse stage, tool goes away from the workpiece, whereas in third case the tool moves towards the workpiece. Their numeral investigation reported on the bubble expansion to the normal direction of tool-workpiece interface taking the shape of hourglass and finally collapses into two parts, considering only EDM process takes place. For the second case, bubble expands to its maximum value causing rapid pressure drop inside the bubble when the tool moves away due to vibration, thus resulting in a rapid vaporization and evacuation of the melted debris. This enhances the MRR. Third case, when tool again moves towards the workpiece, causes the reduction of bubble volume to the smallest one and therefore experiencing shortest lifecycle. Their experimental verification on Ti materials using forged copper electrode in die-sinking EDM shows the increase of MRR with the pulse on time, however, selection of proper process parameters as well as synchronization of vibration frequency and discharge frequency have significant effect on the MRR (Figure 11).

On another study, Shervani-Tabar et al. [43] also investigated the effect of tool and workpiece shape on the bubble dynamics after the occurrence of necking phenomena during UVA-EDM. With the creation of necking, vapor bubble existing in the spark gap splits into two parts, which causes liquid jets to be formed on the upper and lower bubble boundary. The liquid jet on the upper boundary aids in impinging on the tool surface and lower one hits on the workpiece surface. This phenomenon in turn removes the debris from the spark gap, as well as causes the erosion of both electrodes. Since the shape and the velocity of liquid jet can be influenced by the tool and workpiece shape, this study considers three cases such as: flat tool & workpiece, convex tool & workpiece and concave tool & workpiece surface. As per their numerical simulation, the jet velocity on the vapor boundary has higher magnitude and wider shape in case of concave tool & workpiece surface, therefore, increases the MRR for the concave shape surface. Shervani-Tabar et al. [44] also investigated the bubble dynamics and hydrodynamic behaviour of liquid jet for simultaneous vibration of both the workpiece and tool numerically and reported on the bubble generation in the mid distance of spark gap due to the same frequency and amplitude of tool & workpiece. Under this condition, split parts of bubble are the mirror image of each other and they demonstrate similar dynamic behaviour. Simultaneous ultrasonic vibration causes extension of the vapor bubble lifetime before necking happens compared to the vibration assisted tool only. Also, the rate of bubble growth as well as the collapse phase is larger for simultaneous vibration causing the bubble to reach a larger volume than tool vibration. Therefore, the minimum pressure drops inside the bubble causes ejection of material and enhances the MRR. They also reported increased bubble lifetime, higher rate of growth and collapse phase with the increase of both ultrasonic vibration amplitude and frequency. As a result, it also increases the liquid jet velocity that impinges on the surface, thus increasing the MRR.

**Figure 12:**
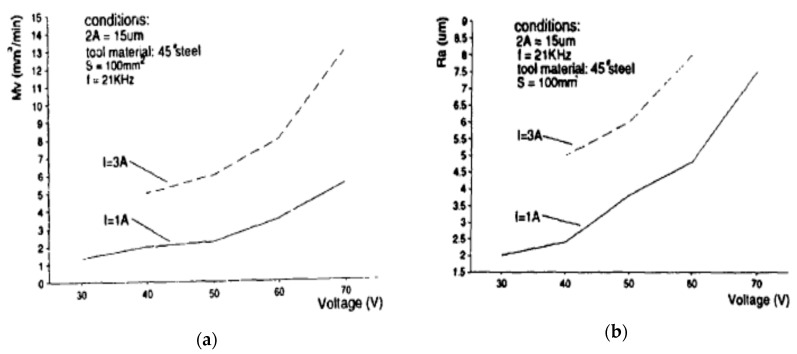
Effect of voltage on (**a**) MRR; (**b**) Surface roughness [45]. (Adapted from [45] with permission—© 1997 Elsevier).

Zhang et al. [45] also reported on the increased MRR and surface roughness with the increase of voltage, current and vibration amplitude during their UV assisted EDM on ceramics with the help of steel tool (Figure 12). This increased MRR can be explained with the effect of better removal of debris, introduction of a new dielectric into the gap, extraction of more liquid materials from the melted pool as well as less re-solidification of liquid metal, as generated by the ultrasonic vibration. Maximum value of vibration amplitude for reaching better machining performance depends on the gap distance between the tool and the workpiece.

**Figure 13:**
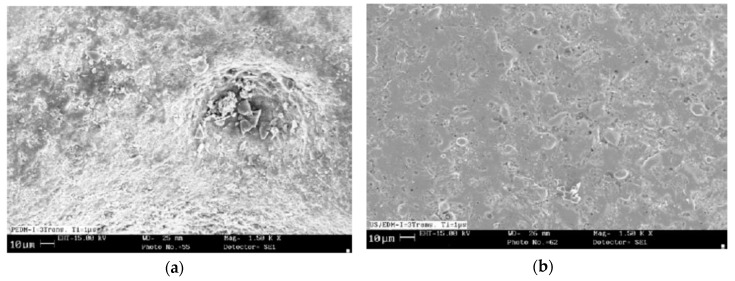
(**a**) Typical SEM micrograph showing pure ED machined surface (I = 11 A. Ti = 1 μs); (**b**) ultrasonic-assisted ED machined surface (I = 11 A. Ti = 1 μs) [48]. (Adapted from [48] with permission—© 2009 Springer).

In order to improve the MRR of insulating ceramics associated with EDM process, Praneetpongrung et al. [46] investigated assistive electrode EDM with the aid of ultrasonic vibration and reported on the effect of tool polarity and ultrasonic vibration amplitude on MRR, EWR, roughness, conductive layer generation. For insulating ceramics like Si_3_N_4_, it is important that the stray conductive layer remains on the ceramics surface to continue the EDM process, however Mohri et al. reported on the disturbance in the generation of this conductive layer due to the tool vibration [47]. Therefore, Praneetpongrung et al. [46] suggested that ultrasonic vibration should be applied to EDM after the transition state in order not to disturb the conductive layer generation. According to their observation, conductive layer does not adhere to the surface when positive polarity is used thus decreasing the MRR significantly, however, the negative polarity can increase the MRR. UVA-EDM with the positive polarity also offers reduced EWR due to the adherence of carbon particles on the tool surface. In addition, vibration amplitude ranging from 2.7–3.5 µm provides higher MRR with lower EWR due to the improved debris evacuation as well as increased normal discharge occurrence. On the other hand, vibration amplitude above 3.5 µm causes decrease in the MRR with the increase of EWR due to the occurrence of the abnormal discharges. Polishing followed by UVA-EDM is recommended for the enhanced surface roughness. Abduallh and Sahabgard et al. [48] have investigated the surface integrity characteristics of tungsten carbide (WC) during UVA-EDM. Roughly 10% increment of surface roughness was observed due to the UVA compared to pure EDM. This increase in surface roughness may be because of the hot spot-core plasma as well as the imposed current, which reduces the ignition delay time and intensive micro-jets. The micro-jets aid not only in terms of improved flushing but also reduced unstable pulses (Figure 13). They also reported on the increased tool wear due to the cavitation effect created by UV assistance. Although UVA-EDM is reported to increase the surface micro-hardness compared to EDM at higher energy condition, hardness value remains unaffected. Surface topography generated by the both processes presented in Figure 13 shows difference in terms of crater size and occurrences of arcing.

**Figure 14:**
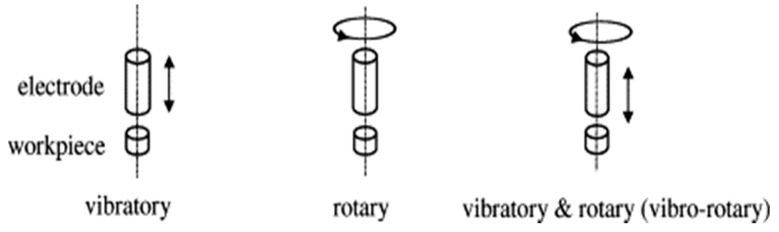
Vibratory, rotary and vibro-rotary electrodes [49]. (Adapted from [49] with permission—© 2002 Elsevier).

Ghoreishi et al. [49] studied the effect of vibratory, rotary and combined vibratory-rotary tool (Figure 14) on the MRR, TWR and surface roughness and reported on the better performance of vibratory tool in terms of MRR and TWR. Also, higher frequency vibration has more pronounced effect on the MRR especially on the finishing zone compared to the lower frequency vibration, as it can eradicate issues such as short circuiting, arcing and pulse instability into the narrow gap condition. For the finishing operation, the high frequency vibration along with the rotary motion can provide better performance compared to rotary only, pure and vibratory only EDM. On the other hand, combined vibro-rotary EDM enhances the MRR by 35% compared to vibro-EDM. However, rough EDM does not require any assistance in terms of improved flushing and can achieve maximum MRR with the higher current setting alone.

In another study done by Lee et al. [50] insulating ceramics Al_2_O_3_ was machined using UVA-EDM and their results suggested increased MRR when compared with the summation of MRR by UVA, EDM, however, increased MRR also enhances generated surface roughness. Increase of the discharge power or voltage, vibration amplitude and frequency aid in MRR due to the efficient discharge distribution and easier debris removal. Uhlmann et al. [51] studied high temperature resistant materials using low frequency vibration (0–700 Hz) for high aspect ratio structure and reported on the leading effect of the vibration amplitude. Their results suggested reduced MRR when applying higher frequency along with the high amplitude due to the short circuiting phenomena. However, the high frequency and low amplitude can provide better machining efficiency due to the better flushing. Their study also reported on the 11% increment of MRR and 21% reduction of tool wear due to the UV assistance [52].

**Figure 15:**
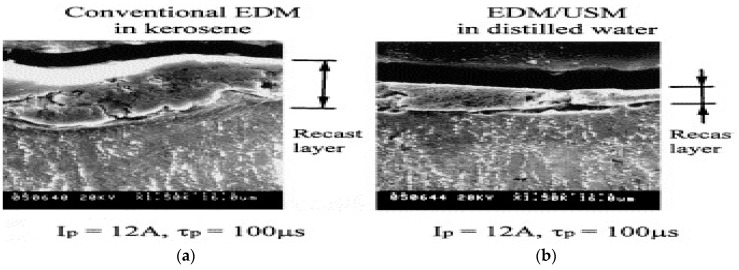
Typical SEM micrographs of the recast layer [53]. (Adapted from [53] with permission—© 2000 Elsevier).

Lin et al. [53] studied the effect of SiC abrasive mixed kerosene and distilled water during UVA-EDM and reported on the enhanced MRR due to the additional removal of materials by abrasive action; however, higher concentration of SiC seems to have negative effect due to the accumulation of abrasive in the spark gap causing the unstable discharges. In general, MRR from UVA-EDM is higher than the EDM due to the larger crater size. In addition, distilled water as dielectric offers higher MRR and lower REWR (relative electrode wear ratio)) compared to kerosene. On the other hand, surface roughness generated by the kerosene appears to be better than distilled water due to the combined effect of higher cooling rate of distilled water and poor thermal conductivity of Ti. In addition, less recast layer is observed in case of distilled water due to its higher cooling rate causing the conduction of less energy into the machined area (Figure 15).

**Figure 16:**
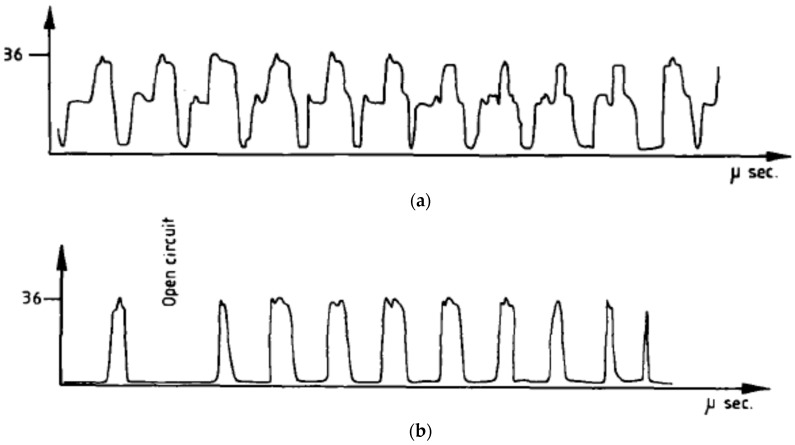
(**a**) Current pulses with ultrasonic vibration (**b**); Current pulses without ultrasonic vibration [55]. (Adapted from [55] with permission—© 2018 Elsevier).

**Figure 17:**
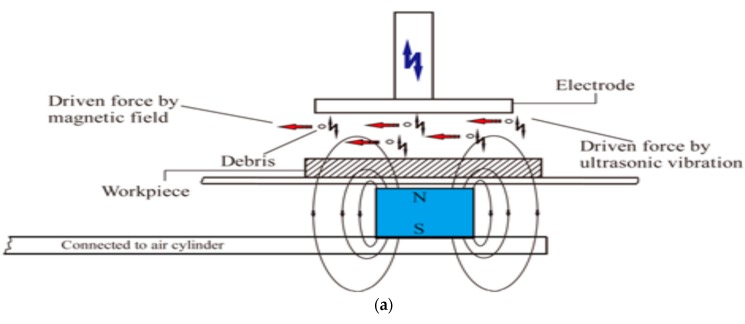

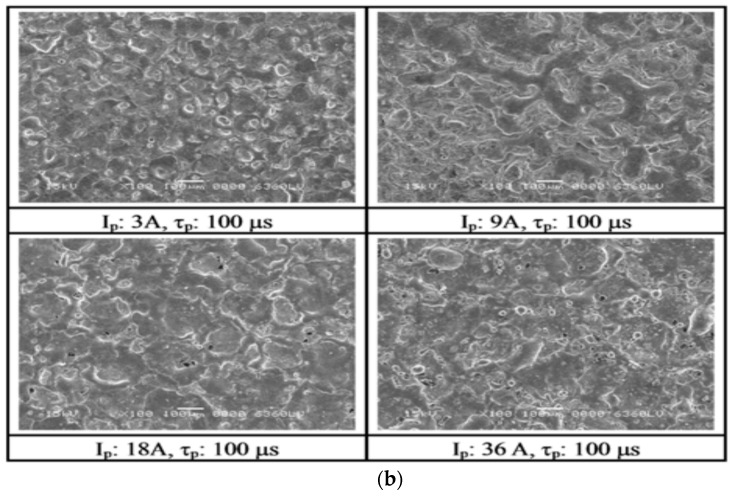
(**a**) Schematic diagram of the driven forces on the debris within the machining gap of the hybrid process; (**b**) Micrographs of machined surface obtained by the hybrid process with various levels of discharge energy [25]. (Adapted from [25] with permission—© 2014 Springer).

Zhixin et al. [12] proposed low cost pulse EDM along with the tool vibration in order to drill deep holes in ceramics materials and their method replaced the pulsed generator, as discharge pulse is generated by the ultrasonic resonant system. Several problems of EDM when drilling deep holes are difficulties in removal of debris, difficulties in complete material removal due to the surface tension, bonding of solid and liquid elements. However, UV assisted EDM can offer better evacuation of debris, thus, reduces metal re-solidification by creating pressure variation in the gap. Their results reported on the enhanced MRR with the increase of voltage due to the higher energy availability and better flushing. Thoe et al. [54] also studied UVA-ED drilling operation on Ni alloy covered with nonconductive coating and reported on the enhanced MRR as well as less heat affected zone along with micro-cracks due to the stable discharge condition. In addition, increase of the current aids in an increased machining depth, however, the tool wear is also equal or double in length. Murthi et al. [55] studied the pulse characteristic during UVA-EDM of high carbon, chromium steel and reported on the insignificant effect of UV on the ignition delay time. However, UV assisted current pulses show different shape compared to the one without UVA (Figure 16). In general, EDM current signal has rectangular shape, whereas UV assisted EDM exhibits pre-breakdown current which might be due to the acousto-electric effect. However, due to its low magnitude density, this current does not have significant effect on machining. Nevertheless, UV-A increases the number of effective current pulses due to the reduction of the short circuiting and arcing. Lin et al. [25] proposed a new hybrid process which combines UVA-EDM with magnetic force assistance and is applicable for large area EDM. Their results reported on the improved machining stability due to the magnetic force assisted debris evacuation (Figure 17) as well as better flushing due to UV assistance. The MRR of this hybrid process increases along with the increase of the peak current as well as pulse duration. However, higher peak current also increases the EWR, whereas increased pulse duration decreases EWR due to the reduced density of spark energy. Also, the tool electrode polarity affects the EWR. When polarity changes from positive to negative, EWR along with MRR reduces a great deal. Their study reported on 34% more MRR and 21% reduced roughness for UVA-EDM than conventional EDM. Figure 17b also presents the machined surface topography using hybrid process. It is obvious from these images that, increase of discharge energy aids in the enlarging and deepening of the crater size which comes with other surface defect such as micro cracks. Therefore, larger discharge energy hinders the surface integrity and to some extent compromises its application capability.

**Figure 18:**
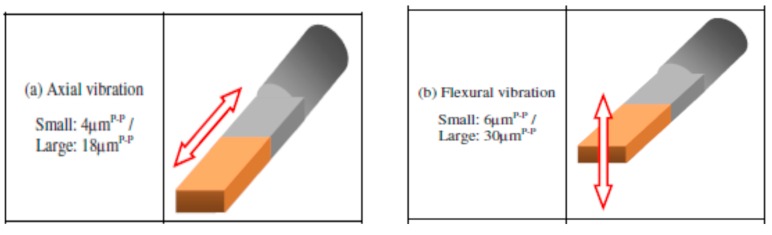

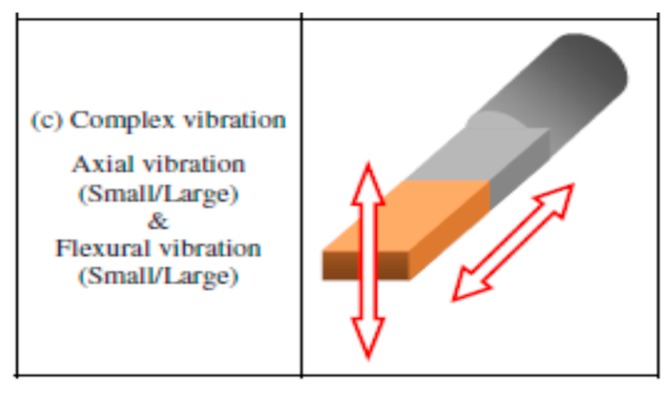
Various types of ultrasonic vibration mode and amplitude [58]. (Adapted from [58] with permission—© 2013 Elsevier).

Srivastava et al. [56,57] compared the performance of normal and cryogenically cooled tools with and without the assistance of UVA-EDM. Their study narrates the enhancement of MRR and EWR with the increase of current and pulse on time. Cryogenically cooled tool without UV-A exhibits lower MRR compared to the other two tools. The reason behind this can be the reduced temperature of cryogenically cooled tool, which requires longer time to get heated allowing less available heat for melting or vaporization. The MRR can be improved with the increase of pulse on time. However, after a certain rise of pulse on time, the MRR shows declining magnitude due to the frequent re-solidification of molten material. In addition, EWR for all three tools increases with the increase of pulse current. Moreover, the surface roughness for the cryogenically cooled tool with and without UV-A exhibits better finish than the normal tool due to the effective debris removal and smaller crater size. However, with the increase of pulse on time and pulse current, MRR as well as surface roughness goes higher. Iwai et al. [58] investigated the machining of PCD(polycrystalline diamond) materials, which contains non-conductive diamond particles and is known as hard to machine material, with the UVA-EDM(ultrasonic vibration assisted) where application of ultrasonic vibration modes varies. Different ultrasonic vibration modes are presented in Figure 18. As per their results, complex mode vibration exhibits 1.7 times better removal than without tool vibration and 3 times better than pure axial vibration. Although complex vibration mode offers higher EWR, insignificant difference in EWR is observed for without vibration and pure axial vibration cases. In addition, surface roughness associated with the complex mode is relatively less than the axial mode vibration, although the difference is not very significant.

**Figure 19:**
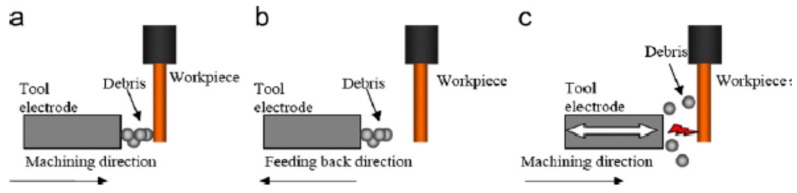
The effect of vibration on the adhesion process. (**a**) Without vibration; (**b**) Feeding back action and (**c**) With vibration [64]. (Adapted from [64] with permission—© 2018 Elsevier).

In order to avoid the environmental hazard of dielectric fluid, researchers proposed dry EDM mechanism, however, this process has limitations in terms of poor surface quality due to the inefficient flushing. Xu et al. [35,59] investigated thermal stress related material removal mechanism during UVA-EDM in gas medium. As per their observation, thermal spalling which depends on the material properties is considered as the removal mechanism. This removal mechanism occurs in four steps, such as: generation of thermal stress, generation of micro-cracks, grain breaking process and particle striping. The debris generated from this process is effectively flushed out by the high-pressure gas flowing through the vibrating electrode. This volumetric material removal is not only by melting but also by cavitation erosion. They also reported on the increased MRR when pulse on time, current and voltage were increased due to effective heat transfer to the workpiece. Opposite trend was observed for the increase of pulse off time due to the inefficient heat transfer. Generation of micro-holes for micro-products also experience challenges associated with the debris evacuation and it becomes a serious issue when the generated micro-holes need to have higher aspect ratio. Therefore, the application of UV to the electrode during micro-EDM process plays a very significant role [60,61]. Jahan et al. [61] compared micro-hole fabrication with and without UV assistance and reported on the higher aspect ratio holes (16.7) for difficult to cut material tungsten carbide with UVA-micro-EDM. Their study also reported on the noticeable improvement in MRR and decreased EWR. This improvement in term of MRR, EWR and dimensional accuracy can be explained by the discharge ratio increment, reduced arcing or short-circuit phenomena, as well as the effect of flushing mechanism [62]. Weiliang et al. [63] conducted a similar experimental study to fabricate arrays of microelectrodes and microstructures using UVA-EDM. Their observation suggested increased MRR with the increase of voltage and increased efficiency of two-fold using 20 kHz frequency compared to pure EDM. They also reported on the cavitation effect created by UV application causing the efficient removal of debris as well as assisting in discharge effectiveness. Mahardiak et al. [64] proposed a micro-EDM monitoring process where, based on the discharge pulse count, discharge energy can be calculated and their result shows good agreement. For the higher MRR, number of pulses increases although average pulse energy remains same. They verified their process monitoring applicability against different process conditions and shape up as well as flat head type machining. They also reported on the reduced machining time for the tool vibration and tool rotation combination compared to the one without tool vibration and rotation. Reduced machining time can be explained with the fact of reduced adherence of debris to the tool electrode due to the tool rotation and vibration, therefore avoiding the occurrence of debris attachment on the tool or workpiece surface, as shown in Figure 19.

**Figure 20:**
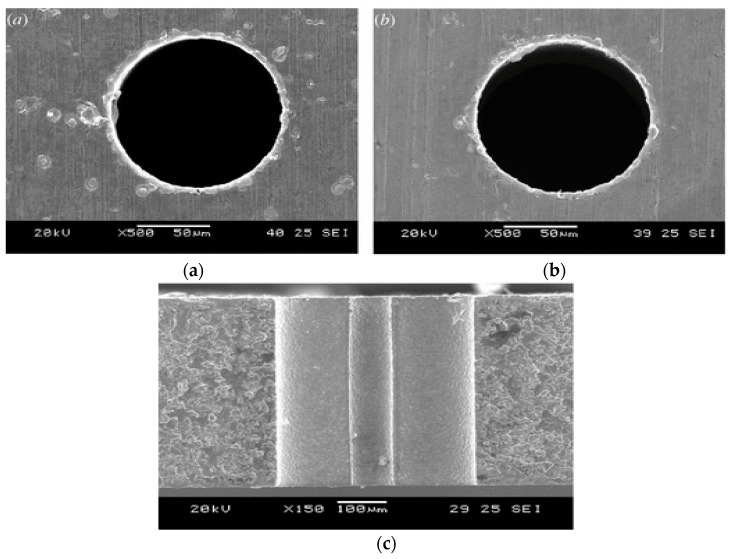
Straight micro hole machined by EDM with capacitance change and ultrasonic vibration: (**a**) hole entrance 117.0 µm, (**b**) hole exit 115.6 µm (**c**) Cross section of a straight micro hole machined by EDM with capacitance change and ultrasonic vibration [65]. (Adapted from [65] with permission—© 2006 Iopscience).

Deep hole fabrication also comes with the taperness effect. Kim et al. [65] used resistance-capacitance (RC) type pulse generator along with UVA-EDM in order to reduce this taperness effect. Their investigation suggested to machine micro-holes as quickly as possible using higher settings of capacitances in order to avoid secondary discharge caused by the debris. The capacitance range of 1000–5000 pF shows insignificant difference between the exit and entrance side of hole. However, increasing machining time always causes overcut of the generated hole. They reported less than 1 µm diameter difference on 500 µm thick steel plate using this method, however when the thickness was increased to 1000 µm, diameter difference increased to 7 µm. Application of UVA can provide not only the reduced machining time but also effective flushing, thus aids in reducing the diameter difference. This new approach recommended use of small capacitance at first and then larger capacitance to achieve straight hole (Figure 20).

Huang et al. [66,67] demonstrated 60% improvement of efficiency by applying UVA while EDM of Nitinol, which is a very difficult to machine material. The amplitude of the ultrasonic vibration can be increased up to a certain value for which the machining efficiency increases, however above that, the efficiency falls again due to the collision of tool and workpiece. In addition, large amplitude affects dimensional accuracy by creating horizontal vibration of the tool. Increase of voltage aids in reduced machining time, nevertheless, provides a higher tool wear. Their study suggested to use a larger tool to increase the machining efficiency with less tool wear effect and to use higher pre-set gap to increase the flushing as well as the machining efficiency. Therefore, by manipulating the gap size, tool size and vibration amplitude, machining efficiency can be improved due to the better stirring effect. Bamberg et al. [68] incorporated tool orbital motion instead of tool vibration while micro-EDM of Nitonol for the hole fabrication. Better surface condition as well less machining time is achieved due to the increased gap between tool and workpiece wall, which reduces the arcing or short circuit phenomena. One advantage of this technique lies in using one single standard tool for creating range of holes due to the orbital effect. Schubert et el [69] narrated 40% process speed increase for metallic material and aspect ratio of more than 5 for ceramics materials due to the application of low frequency ultrasonic vibration to EDM. As per their study, low frequency is particularly advantageous as it does not interfere with the ultrasonic resonant system but is able to create a pressure variation in the dielectric fluid to enhance the speed of fluid.

**Figure 21:**
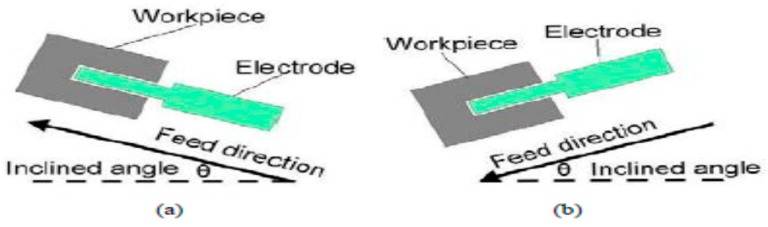
Illustration of inclined feeding (**a**) upward and (**b**) downward [71]. (Adapted from [71] with permission—© 2016 Elsevier).

Mastud et al. [70] investigated reverse UVA-EDM by simulating the debris removal process with the help of Fluent. Their simulation results confirmed the short-circuiting effect due to the debris accumulation and ultrasonic vibration generated compression as well as expansion effect which increases the debris velocity. As per their results, ultrasonic vibration frequency also increases debris particle velocity due to the increased velocity of vibrating tool that transfers the momentum to debris. On the other hand, the amplitude increment increases the pressure variation of the flushing liquid, thus aids in enhancing the debris particle velocity as well. Liao et al. [71] proposed a new inclined micro-EDM drilling with axial vibration application and reported on the enhanced drilling depth compared to horizontal set up due to the associated improved debris removal from the inclined set up (Figure 21). Set up angle of 15° inclination along with the upward feeding of tool gives significantly improved MRR due to the gravity assistance when compared with horizontal set up. 75% increase of drilling depth is reported on Aluminium alloy in this study, whereas, aspect ratio of 26 for 100 µm hole is reported on steel.

**Figure 22:**
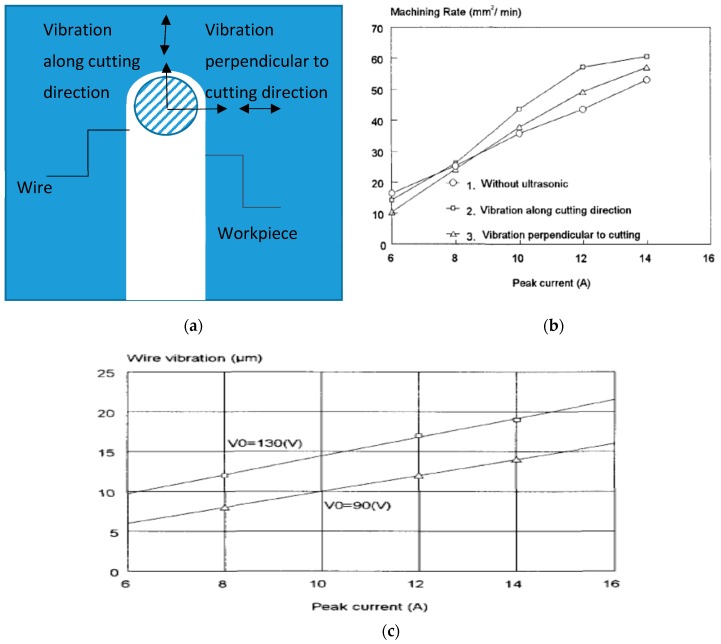
(**a**) Vibration orientation and cutting direction; (**b**) Effect of ultrasonic vibration on the machining rate as a function of the peak current; (**c**) Relationship between the amplitude of wire vibration and the discharge energy [72]. (Adapted from [72] with permission—© 1997 Elsevier).

Other than, EDM, micro-EDM; as well as wire-EDM plays a significant role for hard to cut material fabrication. Several studies reported on the improvement of process by applying vibration assisted WEDM. Guo et al. [72] demonstrated 30% increase in the cutting efficiency as well as reduced residual stress by applying UVA to wire-EDM process. As can be seen in Figure 22a, ultrasonic vibration can be applied both in the cutting direction as well as perpendicular to the cutting direction. Perpendicular to the cutting direction vibration increases the kerf width compared to the vibration applied in the cutting direction. Figure 22b states the increase of cutting rate with the application of UVA. Their study also demonstrated an optimum relation between discharge energy and vibration amplitude for the higher machining rate. Spark gap is a function of discharge energy, that is, increase of energy in general increases spark gap, which can also accommodate larger vibration amplitude. However, if higher amplitude vibration is applied into the lower spark gap, it will result in a short circuit pretty often, whereas, lower amplitude with higher spark gap also does not make much impact on the process. Figure 22c shows the relation between amplitude and current. On another study, Guo et al. [73] also reported on the benefits of using higher frequency vibration that may cause multiple channel of discharge resulting in a better surface as well as a higher machining rate. Higher frequency also aids in reduced wire breakage due to the shifting of discharge point. Crater shape changes from rounder shape (WEDM) to elliptical shape (UVA-WEDM) due to the shifting of discharge channel. In addition, optimum distribution of uniform discharge is achieved with UVA-WEDM, thus also enhances the energy magnitudes to 15% more than the normal the WEDM. Kavtaradze et al. [74] also suggested applying vibration to wire during EDM process to generate the superposition of vibration through numerical investigation. Lipchanskii also reported on improved process performance with the application of UVA to WEDM [75].

**Figure 23:**
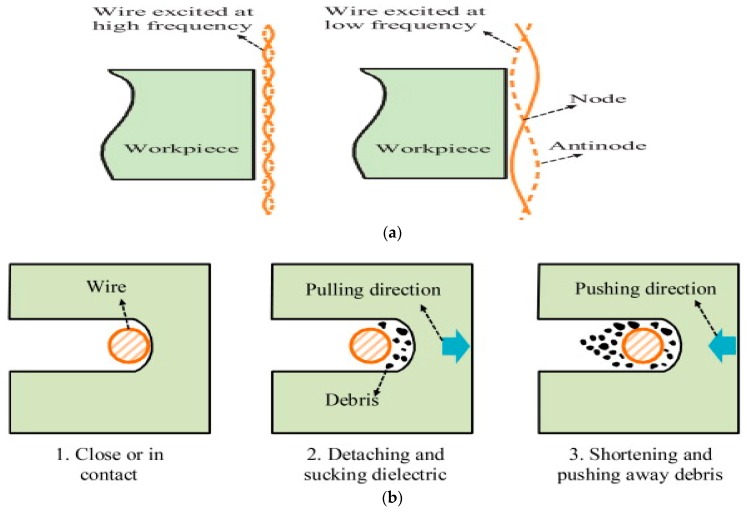
(**a**) Wire vibration state at different frequencies; (**b**) Circulation of debris ejection with vibration of the workpiece [76]. (Adapted from [76] with permission—© 2013 Elsevier).

Hoang et al. [76] investigated on the UVA-WEDM, where the ultrasonic vibration is applied on both tool and workpiece. Their study suggested the generation of nodes and antinodes under the influence of ultrasonic vibration and their number increases with the increase of vibration frequency as seen from Figure 23a. Ultrasonic vibration applied to the workpiece creates a larger pressure variation due to its larger area than wire and therefore, enhances the debris flushing significantly more than when vibration is applied to wire (Figure 23b). Thus, the cutting rate for vibrating workpiece offers 1.5 times more than vibrating wire and 2.5 times larger than the pure WEDM. Although the surface finish gets better for both the tool and workpiece vibration mode, workpiece vibration provides better surface roughness compared to wire vibration due to the difference in the forced wire displacement along the node and antinode direction.

**Figure 24:**
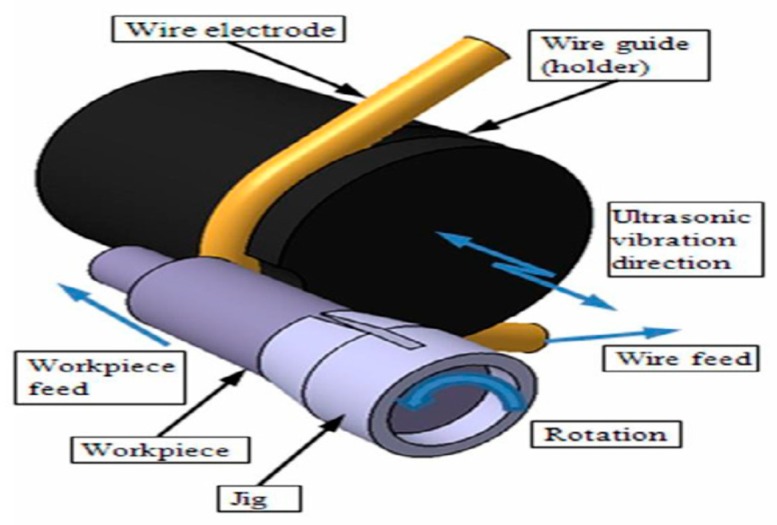
Positioning of the wire guide and workpiece [77]. (Adapted from [77] with permission—© 2013 Elsevier).

**Figure 25:**
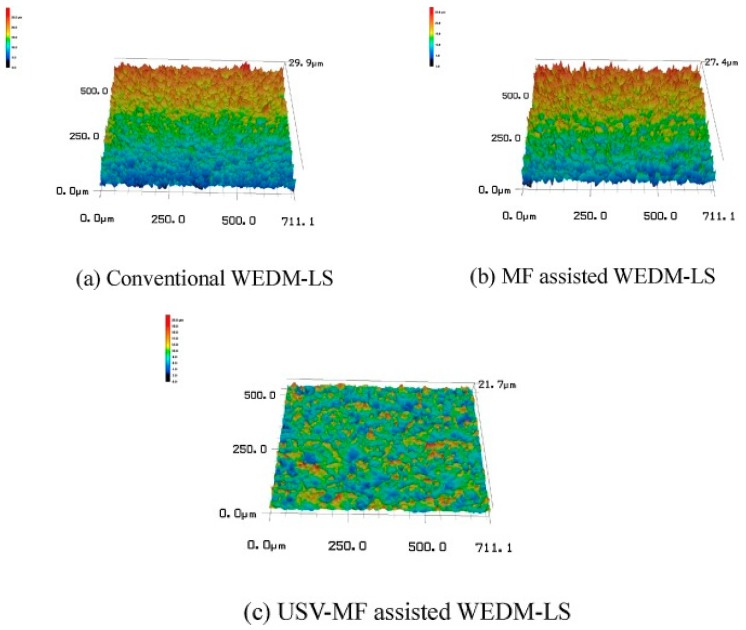
3D micron-scale surface topology of experimental results using three different methods at the same machining parameters (pulse-on time 5 μs, pulse-off time 5 μs, peak current 9A) [79]. (Adapted from [79] with permission—© 2018 Elsevier).

Mohammadi et al. [77,78] conducted UV assisted WED-turning (Figure 24) and investigated the effect of power, pulse off time, vibration, workpiece rotation on MRR using ANOVA method. Their study reported on significant improvement in the MRR due to the better flushing, the erosion mechanism involved with the UV application and reduced wire breakage. This improvement is valid for both roughing and finishing condition. Increase of workpiece rotation results in a higher MRR due to the cavitation effect as well as improved flushing. On the other hand, decrease of pulse off time, causes short circuiting effect due to the improper debris flushing from the interelectrode gap. Both higher MRR and lower surface roughness can be obtained by using low power input with high rotation along with high pulse off time, not to mention the UV assistance. Wang et al. [79] presented a novel method of machining TiNi-01 by combining ultrasonic vibration (UV), magnetic field (MF) and WEDM. UVA and MF was applied together and separately to WEDM-LS process to alleviate debris evacuation from the gap. Uniform distribution of the discharging point induces reduced wire electrode break. Optical profilometer images exhibit positive impact of the hybrid method on the surface roughness (Figure 25).

**Figure 26:**
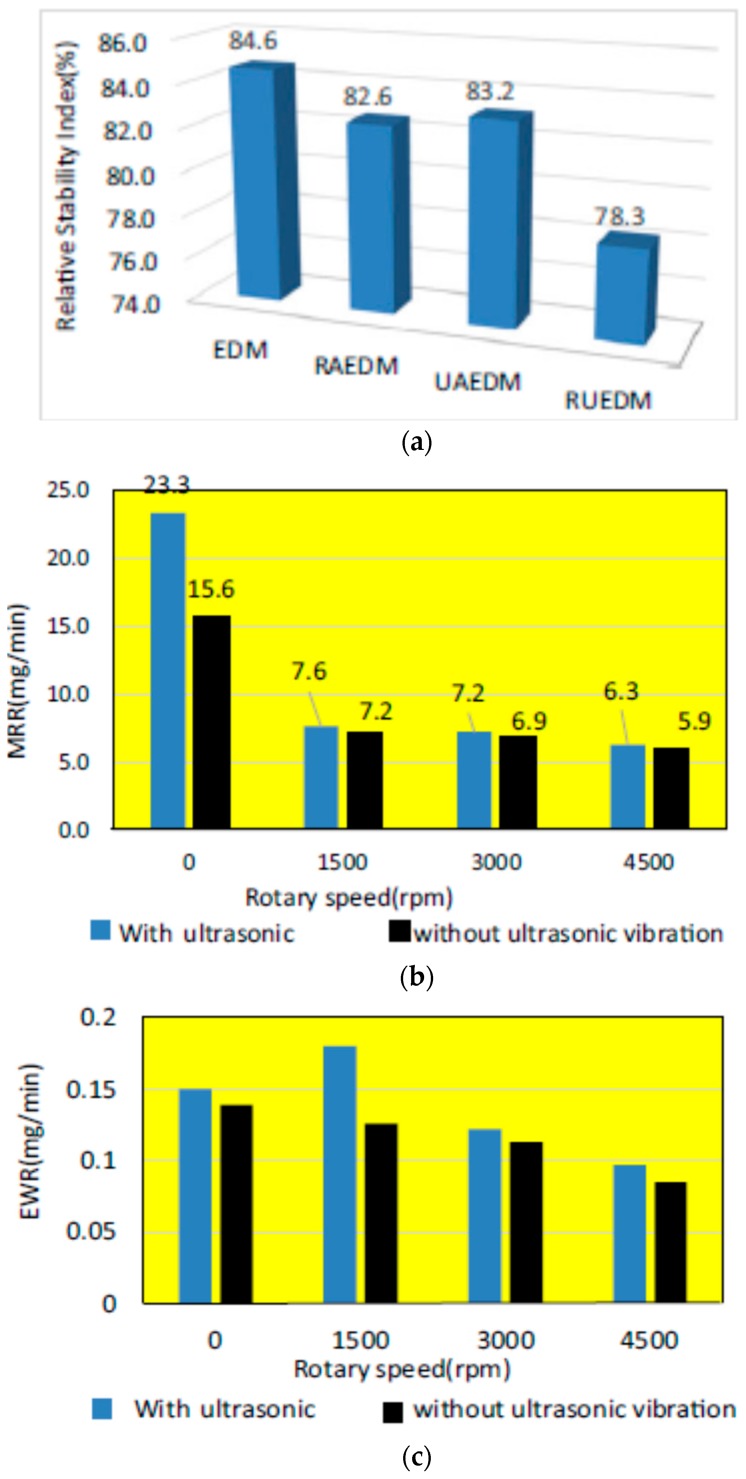
Comparison on relative stability index of various types of EDM; (**b**) Material removal rate affected by various rotation speeds with and without ultrasonic assistance; (**c**) Tool wear rate affected by various rotation speeds with and without ultrasonic [80]. (Adapted from [80] with permission—© 2018 springer).

Wen-Jeng Hsue1 [80] investigated ultrasonic assisted EDM (UVA-EDM), rotary ultrasonic EDM (RU-EDM), rotary EDM (R-EDM) and their effects on the tool wear and process stability where pure EDM process is found to have higher process stability index compared to other (Figure 26a). With UVA, 49% increment of the MRR was reported compared to the EDM process, however, tool wear is affected negatively with the application of UVA (Figure 26b,c). On the other hand, Rotary ultrasonic EDM experiences relatively low process stability compared to other processes due to the combined effect of ultrasonic vibration and rotation. In addition, tool rotation appears to be negatively impacting the MRR compared to the non-rotation condition (Figure 26). Muttamara et al. [81] performed investigation for improving the surface quality by using powder (TiN) mix UVA-EDM. Their study reported on the uniform TiCN layer generation with this process. It was reported that 6 A current with 50 µs pulse on time and 21 A current with 50 µs pulse on time can reduce crack generation on the surface. Frequency of ultrasonic vibration appears to have an insignificant effect on the surface roughness generation. Table 2 provides a summary of the research conducted in UV application of tool.

### 4.2. Ultrasonic Vibration on Workpiece

**Figure 27:**
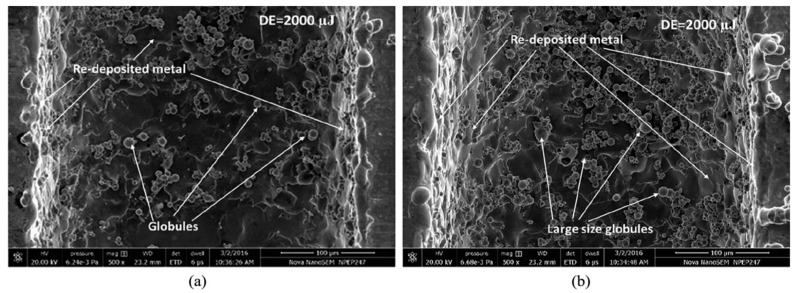
Effect of workpiece vibration on microchannel surface quality (**a**) without vibration and (**b**) with vibration of 160 Hz [84]. (Adapted from [84] with permission—© 2018 Taylor & Francis).

Unne et al. [83] studied on Inconel alloy using low frequency UV assisted micro-WEDM and optimized the input parameters with the help of Box-Behnken design. As per their optimization result, capacitance had the most leading effect on MRR and kerf width; and their study also reported on 66.20% enhancement of MRR with the low frequency UV assisted micro-WEDM. Kerf dimension is influenced by the gap voltage, capacitance and ultrasonic vibration frequency. Low frequency vibration enhances the process performance due to the better flushing as well as less adhesion between the tool/workpiece surface. They conducted a similar study using low frequency UV assisted micro-ED milling and reported on the enhanced MRR with the reduced frontal tool wear. The reason for this enhancement might be because of the following fact: low frequency vibration results in the oscillation of both the dielectric and debris, thus increasing the new dielectric supply to the interelectrode gap. SEM image of the machined channel stated the presence of a greater number of globules when the discharge energy increases. Also, the application of ultrasonic vibration for same level of discharge energy increases this globules due to the rapid cooling of melted materials with the aid of enhanced dielectric circulation compared to the non-vibrating one [84] (Figure 27).

**Figure 28:**
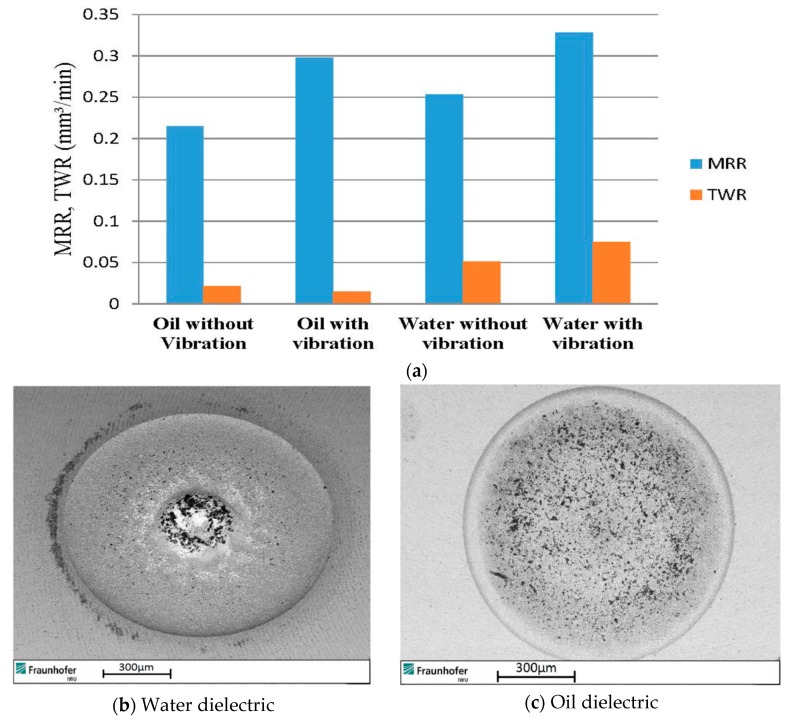
(**a**) Effect of dielectric fluid and vibration on MRR and TWR (vibration amplitude = 6.4 µm); (**b**,**c**) SEM images of machined surfaces [86].

**Figure 29:**
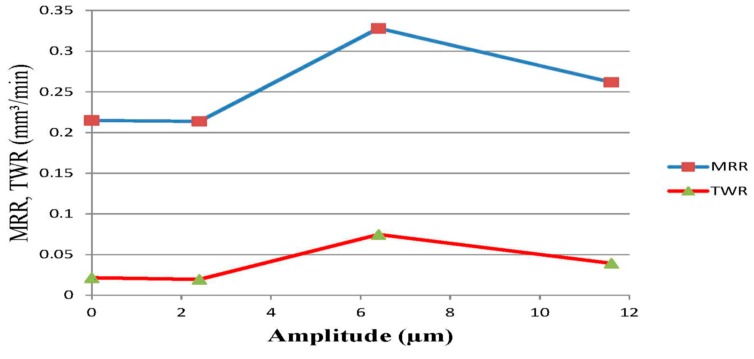
Effect of amplitude on MRR and TWR in oil dielectric machining [86].

Prihandana et al. [85] also conducted an experimentation on UVA-EDM where ultrasonic vibration is applied to the Steel workpiece and their results suggested an improved MRR, reduced tool wear as well as reduced surface roughness. During the workpiece vibration, downward movement of the workpiece is introducing a new dielectric, whereas the upward movement aids in the debris removal. Since the workpiece area is larger than the tool, debris removal effectiveness is enhanced with the movement of workpiece much more. They also investigated on the application of low frequency (300, 400, 600 Hz) and amplitude of vibration (0.75, 1 µm). Their investigation reported on the higher MRR for 1 µm amplitude with 400 Hz than 600 Hz frequency due to the decreased machining stability which resulted in a longer machining time. On the other hand, the tool wear rate was found to be lowest at 300 Hz with 0.75 µm amplitude and highest at 600 Hz. Moreover, the surface roughness can be reduced using a low amplitude ultrasonic vibration compared to the normal EDM, however, the high amplitude tends to increase the roughness. Wamia et al. [86] conducted a similar experimentation on AlSiC metal matrix composite using low frequency ultrasonic vibration (900 Hz) for various dielectrics such as distilled water and oil. Their experiment reported on the higher MRR for distilled water compared to oil, whereas the surface roughness showed better result for oil (Figure 28). In addition, the TWR also increases with the application of ultrasonic vibration when using water as a dielectric. Moreover, dimensional accuracy is improved irrespective of any dielectric. As per Figure 29, MRR and TWR both increase with the increasing amplitude up to a certain magnitude, after that it shows declining values of parameter due to the increased frequency of short circuiting.

**Figure 30:**
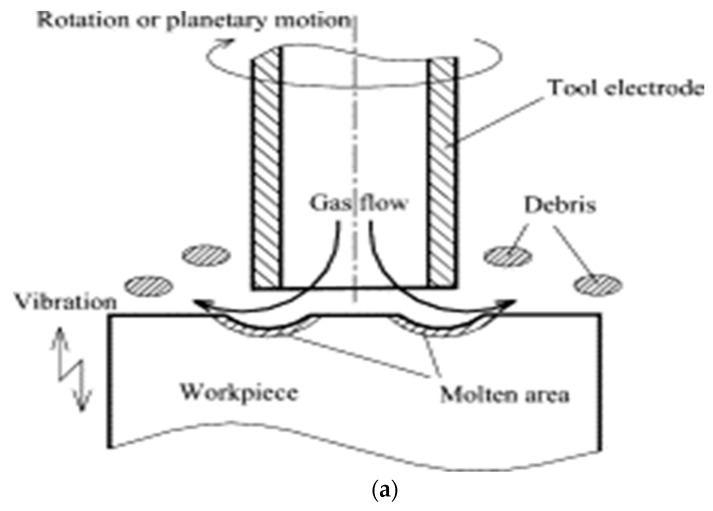

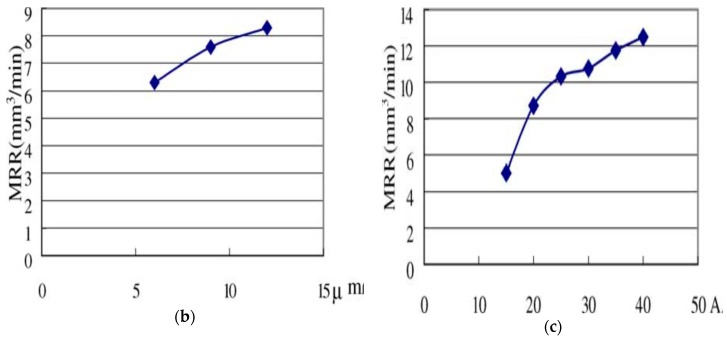
(**a**) Principle of UEDM in gas (**b**) The effect of amplitude of ultrasonic vibration on MRR. (**c**) The effect of discharge current on MRR [30]. (Adapted from [30] with permission—© 2004 Elsevier).

Jiang et al. [87] developed a new vibration platform to avoid energy loss associated with the ultrasonic vibration as well as heat effect and conducted micro-hole fabrication experiments on steel workpiece. Their results reported on five-folds increase of machining efficiency with reduced wear of tool and the reason might be the improved debris evacuation. They also reported on significantly reduced perforation time with this new voice coil motor-based platform. Shabgard et al. [88] also experimented on AISI (American Iron and Steel Institute) tool steel workpiece using variation of pulse on time with two current settings. Their comparative analysis shows, threefold increase of MRR for smaller pulse on time with low current value due to the better debris removal as a result of pressure variation in dielectric caused by ultrasonic vibration. Also, UVA reduces the number of short circuiting, therefore, increases normal discharge frequency. However, surface roughness presents a higher value compared to pure EDM and its value increases with the increase of pulse on time that enhances craters size. Shabgard et al. [89] also did a similar investigation on FW4 welded metal. Their study reported on a higher MRR for smaller pulse on time for ultrasonic vibration assisted EDM, however, pure EDM condition shows increasing MRR trends with increasing the pulse on time. This enhancement for UVA-EDM is due to the better debris removal by upward movement of workpiece and due to better flushing by the downward movement of workpiece. Also, TWR shows higher value for vibration assisted EDM due to the cavitation effect. This TWR value is comparatively lower when the pulse on time is low compared to pure EDM. In addition, due to improved flushing of debris, UVA-EDM presents a higher roughness of surface due to the bigger crater size. Zhang et al. [30,31] proposed UVA-EDM in gas medium where high pressure gas exited from the hollow tool electrode and UV was applied to the workpiece (Figure 30). Their study observed the dependency of MRR on several parameters, such as voltage, discharge current, pulse on time, vibration amplitude (Figure 30b,c). However, surface roughness is mostly influenced by current and pulse on time, whereas the voltage is found to have minimum effect on it. Moreover, they also observed UVA-EDM in gas improved the MRR almost twofold compared to EDM in gas without vibration, however, pure EDM in kerosene still offers higher MRR than EDM in gas medium with ultrasonic vibration.

**Figure 31:**
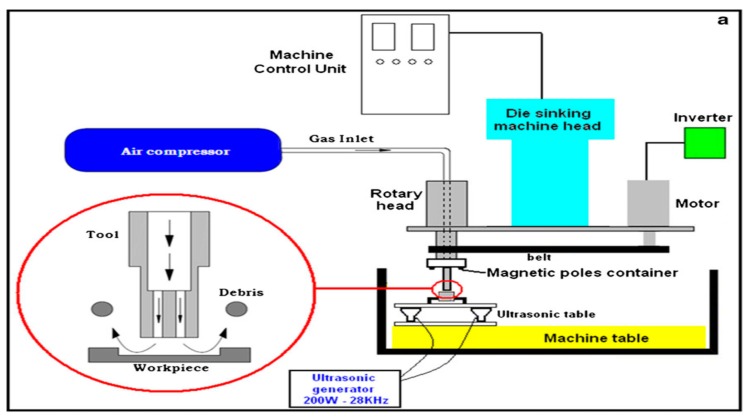
Schematic of Dry EDM setup with rotating magnetic field [90]. (Adapted from [90] with permission—© 2013 Springer).

**Figure 32:**
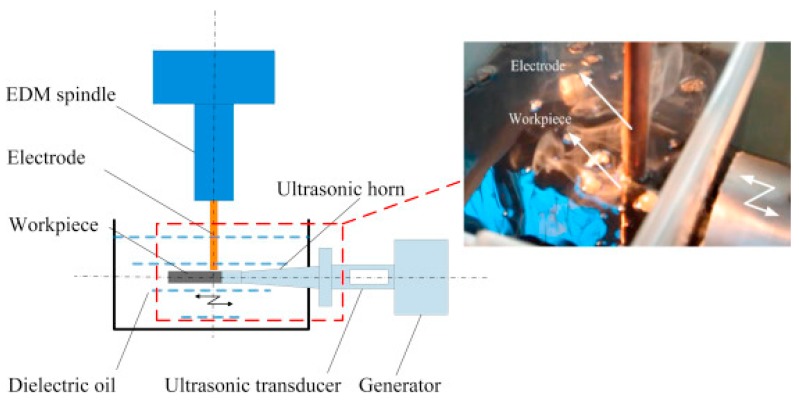
Schematic of horizontal UVA-EDM setup [92]. (Adapted from [92] with permission—© 2016 Elsevier).

Teimuri et al. [90] studied dry UVA-EDM process with the assistance of rotating magnetic field that was generated by rotating magnetic disc attached to the tool holder containing four magnets (Figure 31). Rotating magnetic field aids in debris removal; thus, offers improved process stability in terms of higher MRR as well as better surface. However, it has negative effect on hole overcut due to the acoustic cavitation and tool wear caused by reduced ignition delay. As per their results, brass electrode offers higher MRR compared to the copper electrode due to its higher electrical conductivity. However, it also experiences more EW and roughness due to its low melting temperature. Their proposed regression models for MRR, EWR and surface roughness show a good agreement with the experimental results. Qinjing et al. [91] also proposed another new method combining EDM and UVA mechanical machining of polycrystalline diamond workpiece using a bronze bonded diamond grinding wheel. Ultrasonic vibration amplitude applied during the process is found to have a greater effect than the frequency and it aids in an improved debris removal. They also reported on the increase of MRR with the increase of pulse on time. For given pulse on and off time, an increasing current also increases the processing speed. Increase of pulse on time also enhances the MRR up to a certain critical point due to the increased single pulse energy, then MRR falls again due to the transference of heat to the electrode and workpiece rather than the erosion purpose. Che et al. [92] proposed horizontal UVA-EDM and reported on the three fold increment of MRR as well as 20% enhancement of process accuracy due to the effective heat transfer compared to the traditional EDM. The process setup is presented in Figure 32. They also reported on the better surface roughness due to the improved debris evacuation caused by the horizontal vibration.

**Figure 33:**
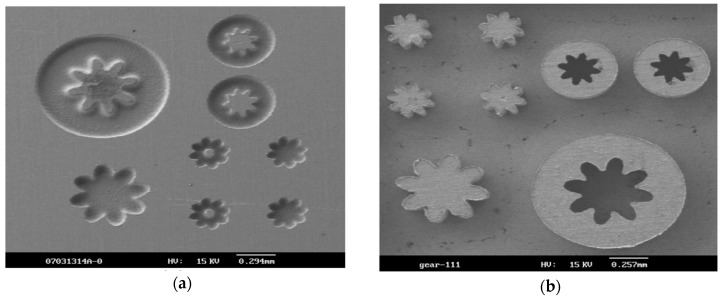
(**a**,**b**) SEM photographs of micro-gear-array electrodes [20]. (Adapted from [20] with permission—© 2008 Elsevier).

Fabrication of non-circular micro-structures always has difficulties in terms of debris evacuation due to narrow discharge gap, therefore, machining efficiency can be significantly low. Tong et al. [20] applied UVA-EDM while fabricating non-circular microstructures on steel. Their results reported on the improvement of efficiency and discharge stability due to the improved fluidity of dielectric caused by the alternating pressure wave created by ultrasonic vibration. They also achieved 18 times higher machining efficiency as well as an increased dimensional accuracy of 10.5 μm, compared to the pure EDM condition by applying 6 kHz ultrasonic vibration with 3 μm amplitude. Figure 33 presents fabricated micro gears using UVA-EDM.

**Figure 34:**
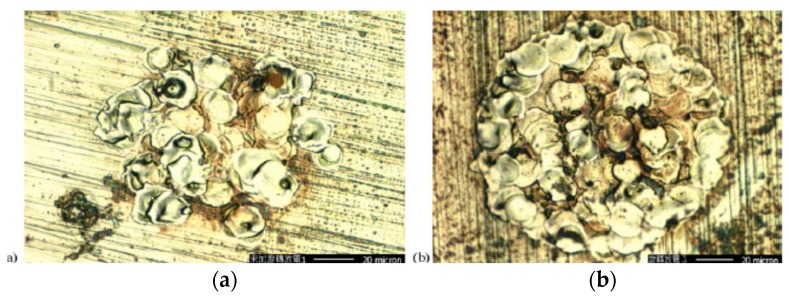

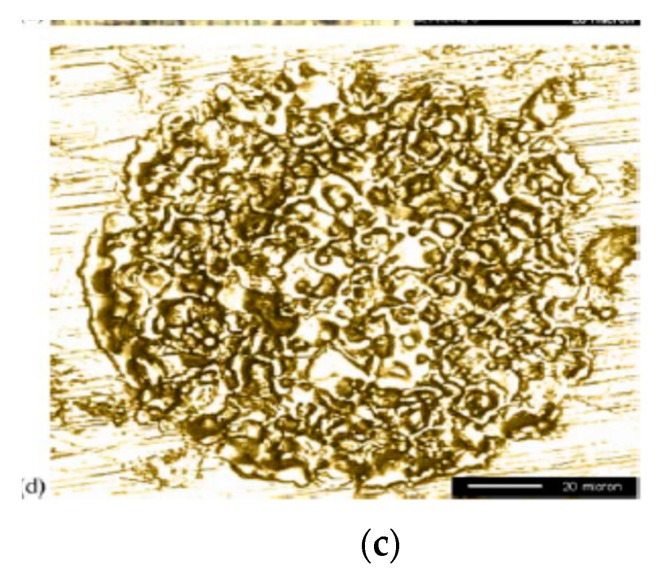
Image of surface (**a**) without rotation & vibration (**b**) with tool rotation (**c**) with rotation & vibration machined surface by micro-EDM [95]. (Adapted from [95] with permission—© 2006 Elsevier).

Hao et al. [93] proposed to investigate high frequency UVA-EDM in order to fabricate 3D mould cavity and reported on the improved process stability as well as discharge ratios due to the increase of favourable discharge gap. Maximum 6.5 times increase of machining efficiency was reported for 5 kHz frequency and 2.7 µm amplitude, for which the MRR achieved was 1.4 × 105 µm^3^ s^−1^. The machining depth increases with the increase of amplitude and frequency, where frequency is the most dominant parameter. In addition, slower scanning speed also enhances the dwelling time in the favourable discharge range, therefore, aids in improving the discharge ratio as well as stable machining. Fu et al. [94] developed a new piezoelectric self-adaptive micro-EDM process. Its working principle is based on inverse piezoelectric effect which is advantageous in terms of evacuation of debris, lowering short circuiting phenomena and accommodating self-elimination of short circuit. Their study reported on the better process stability as well as reduced machining time with this new technology. Due to reduced short circuiting, EWR is also found to be reduced. Chern et al. [95] reported an increased MRR with the increasing voltage and a significant enhancement in the MRR compared to only EDM condition for different tool electrode materials. Chern at al considers four different cases, such as no rotation and vibration, only rotation no vibration, only vibration no rotation, both vibration and rotation and conducted micro-EDM on alloy steel (Figure 34). Their results suggested that without both ultrasonic vibration and tool rotation, surface generated is of very poor quality (Figure 34a), whereas, tool rotation even without ultrasonic vibration improves the surface condition comparatively (Figure 34b). Best surface condition can be seen when both tool rotation and ultrasonic vibration are applied as a result of efficient coolant circulation due to the combined effect of vibration and tool rotation (Figure 34c).

**Figure 35:**
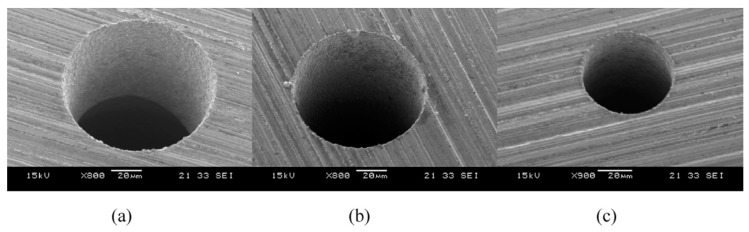
(**b**) Fabrication of micro-electrodes using on-machine fabricated microelectrode; (**a**) micro-hole of Ø 100 m, aspect ratio 5 using vibration with (K_v_ < 1), (**b**) micro-hole of Ø 75 m, aspect ratio 10 with vibration of (K_v_ > 1), (**c**) micro-hole of Ø 60 m, aspect ratio ~17 with vibration of (K_v_ > 1) (SEM images are taken at 30° tilt angle) [23]. (Adapted from [23] with permission—© 2012 Elsevier).

Jahan et al. [23] derived a numerical model for understanding the low frequency vibration applied to the workpiece during UVA-EDM of tungsten carbide. Their study reported on the performance improvement particularly at lower discharge energy due to the continuous change of discharge gap, thus allowing the fresh liquid to enter and evacuate the debris frequently. Stable machining process is reported due to the increased discharge ratio and reduced short circuiting. In addition, reduced short circuiting phenomenon also helps in obtaining a better rim surface as well as dimensional accuracy. They also reported on micro-hole of 17 aspect ratio with 60 µm diameter on WC with this approach (Figure 35).

**Figure 36:**
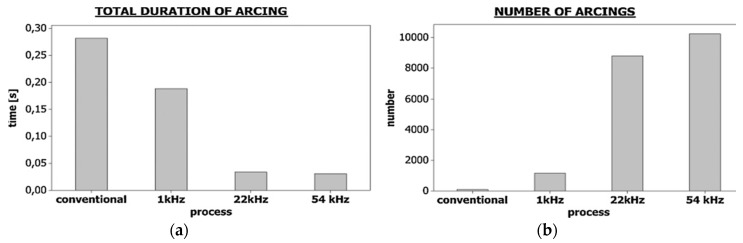
Total duration and number of arc discharges [22]. (Adapted from [22] with permission—© 2011 Elsevier).

Garn et al. [22] studied micro-EDM with the UV assistance to the workpiece and observed an initial delay which is a function of tool diameter, hole geometry and spark energy while machining of deep micro holes. The reason behind this initial delay is found to be arcing which results in the retraction of the tool from the workpiece which leads to the open circuit process. They also reported on the increase of short circuit numbers and decrease of their duration with the application of UVA (Figure 36). Therefore, applied UVA can enhance the machining performance by reducing the machining time.

**Figure 37:**
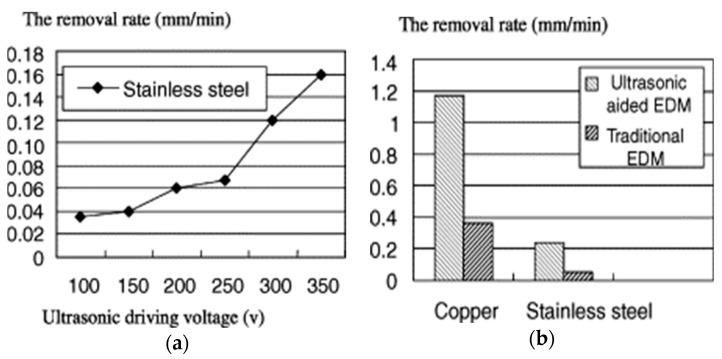
(**a**) The relationships between the removal rate and the ultrasonic driving voltage. (**b**) removal rate and different workpiece materials [96]. (Adapted from [96] with permission—© 2003 Elsevier).

Gao et al. [96] reported micro-EDM process of stainless steel while ultrasonic vibration was applied to the workpiece and achieved eight folds increase of efficiency for 0.5 mm thick workpiece compared to the traditional EDM. Workpiece vibration also enhances the aspect ratio of hole generated on tungsten workpiece. They also observed the increase of MRR with the increasing voltage. In addition, MRR increases with the application of UV is more pronounced for copper than stainless steel (Figure 37).

Prihandana et al. [97] also investigated powder mixed dielectric with and without ultrasonic vibration applied to the workpiece during micro-EDM. Effect of conductive graphite powder in dielectric without vibration increases the spark gap and enhances the debris flushing from interelectrode gap, therefore, improved machining with a smoother surface is possible to generate. However, there is an optimum magnitude of powder concentration which is 10 g/L above which, the machining stability is negatively affected due to the short circuiting caused by the excessive powder deposition on the surface. Now with the application of both vibration and powder (20 g/L), the machining time is reduced significantly. The reason behind the improved machining time might be the efficient discharge frequency due to the powder and better debris flushing due to the ultrasonic vibration. Lowest surface roughness can be achieved for 15 g/L powder concentration, where the craters generated are much well defined and circular shape than those generated during pure EDM. Sundaram et al. [98] investigated UV assisted micro-EDM process using Taguchi design of experiment for A2 tool steel in order to optimize the process for higher MRR with the lowest EWR. Their results reported on a higher MRR when used 60% peak power with 3300 pF capacitance. The machining time is found to have a leading effect on tool wear, therefore increased tool wear with the increase of machining time is observed. Kumar et al. [99] also investigated on EDM of AISI 1045 steel workpiece using low frequency ultrasonic vibration and observed better debris removal due to the ultrasonic vibration thus improving the surface roughness. By analysing Signal to Noise (S/N) ratios, the cutting speed is found to be influenced by pulse off time mostly and least influenced by voltage. However, for surface roughness, wire tension is the leading factor and frequency is the least significant factor. Table 3 provides an overview of EDM researches using UV assisted workpiece.

### 4.3. Ultrasonic Vibration on Dielectric

**Figure 38:**
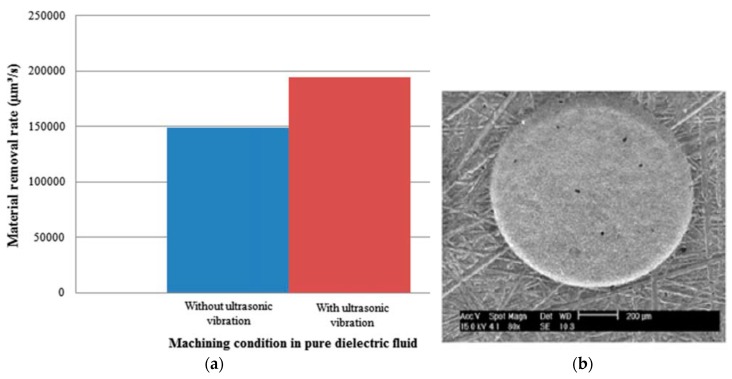
(**a**) Comparison of material removal rate for Cu as a workpiece and brass as tool electrode for machining condition in pure dielectric fluid; (**b**) Machined surface image of Cu–W surface with brass as the tool electrode obtained by m-EDM processing, using MoS2 powder with ultrasonic vibration of dielectric fluid and a powder concentration of 2 g/L [21]. (Adapted from [21] with permission—© 2009 Elsevier).

The idea of applying ultrasonic vibration on the dielectric fluid during micro EDM process is presented by Prihandana et al. [21,103]. The ultrasonic vibration is applied to raise the kinetic energy of discharge debris with a frequency of 43 kHz. According to the result of the experiment, UV application to dielectric containing MoS_2_ powder, showed a considerable increase in material removal rate (Figure 38a) and likewise surface quality improvement due to the reduced adhesion as well as non-deposition tendency of powder particles. Frequency of discharge is enhanced by the presence of micro-particles, the density of which provides a strong impact causing the MRR enhancement. In addition, particles suspension on the surface distributes the current evenly on the surface and aids in reduced black spots. Moreover, lubricity of these micro-particles aids in improved surface finish.

**Figure 39:**
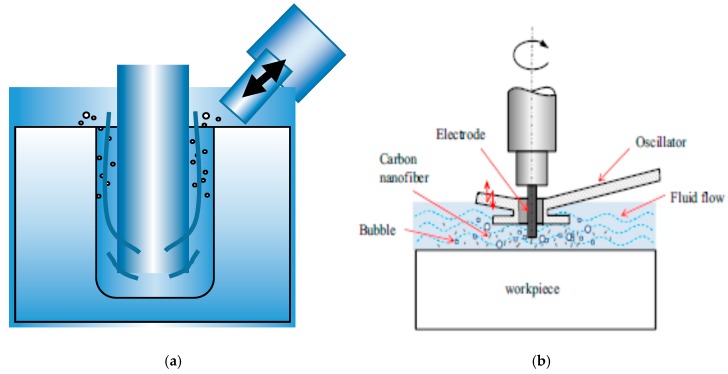
(**a**) Ultrasonic sonotrode in micro EDM [69]; (**b**) Schematic diagram of the proposed hybrid micro-EDM [100]. (Adapted from [100] with permission—© 2014 Elsevier).

Wire EDM study with the assistance of ultrasonic vibration induced cavitation effect is presented by Ghiculescu et al. [104] which shows the enhanced machining rate due to the cavitation effect. This effect was generated by the longitudinal oscillations of an acoustic chain that is submerged in liquid. Dielectric liquid holds the position between the electrode and workpiece and it is contained in the hopper without compromising the precision level. A similar research was also reported by Schubert et al. [69]. Their study suggested the immersion of sonotrode in the dielectric liquid. Ultrasonic vibration of sonotrode can be organised to reach its high intensity at the machining zone. As a result, it rises the speed of the operation. This process is illustrated in Figure 39a. Due to the applied ultrasonic vibration to dielectric, tool electrode also experiences some kind of excitation as the sonotrode orients in a non-coaxial direction. During the process, cavitation effect due to the nearfield vibration occurs, which is further enhanced by the debris particles generated by EDM process within the gap. The gas bubble of cavitation effect can aid flushing process by transporting debris out of the spark gap. This study also reported 5% process speed improvement due to the application of 60° angle ultrasonic vibration to dielectric using sonotrode.

**Figure 40:**
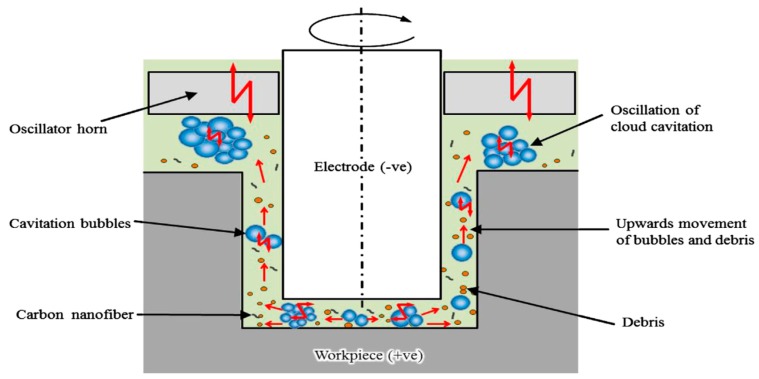
Schematic model for debris removal through the cavitation assisted micro-EDM of a deep micro-hole [100]. (Adapted from [100] with permission—© 2014 Elsevier).

Another similar investigation was conducted by Ichikawa and Natsu [102] using micro ED-Drilling process. They also studied the effect of ultrasonic vibration on the dielectric liquid. Their results demonstrated a small tool wear ratio, shorter machining time and reduced lateral gap between both electrodes due to the application of UV. In addition, UV also facilitates the micro ED-Drilling using a smaller discharge energy which faces certain challenges to remove the debris due to the low spark gap associated with the low discharge energy. Thus UV assistance can improve this debris removal condition. Muttamara et al. [81] performed investigation for improving the surface quality by using UVA-EDM with TiN powder mixed dielectric liquid. The copper tool electrode was used for surface fabrication on SKD11 steel. Machined surface is found to contain debris, cracks, craters and voids. Nevertheless, cracks generated can be reduced by the TiN powder deposition. Higher discharge current can reduce the cracks whereas lower discharge current can reduce the pores. Ultrasonic frequency does not seem to be affecting the surface roughness significantly. Liew et al. [100] used probe-type vibrator to implement the ultrasonic vibration to the dielectric fluid in micro-EDM process (Figure 39b). The application of UVA on ceramic materials for making micro holes using different machining parameters were investigated by the authors. Experimental results showed strong dependency of machining characteristics on the amplitude of ultrasonic vibration and maximum hole depth is achieved for 10 µm amplitude. UVA causes stirring, as well as cavitation effect, which combinedly impedes the deposition of tool material. Stirring effect aids in an uniform distribution of carbon nanofibre in dielectric, whereas the cavitation aids in flushing out the debris. Cloud cavitation exists not only on the surface but also on the further deeper zone and its upward as well as oscillation flow aids debris removal effectively (Figure 40). Both carbon nanofiber and ultrasonic cavitation facilitates effective machining of micro-holes with more than 20 aspect ratio with 10 µm diameter in a short period of time.

In another similar study, Liew et al. [101] investigated reaction bonded silicon carbide material using tungsten tool and studied the influence of ultrasonic vibration amplitude, tool geometry, as well as working distance. Due to the presence of carbon nanofiber in dielectric, improved MRR as well as reduced tool materials deposition were found. In addition, the hemispherical shape tool offers better performance compared to the flat head tool due to the improved debris evacuation during low voltage. However, during high voltage condition, the formation of fewer dimples without protrusion was observed due to the improved flushing. On the other hand, reduced distance between the workpiece and ultrasonic unit provides an enhanced cavitation effect, therefore results in a smooth surface with less tungsten deposition. Higher vibration amplitude results in a lower tool material deposition on the workpiece.

## 5. Effect of Ultrasonic Vibration on EDM Performance Parameters

Researchers have been investigating the influence of ultrasonic vibration on micro-EDM, wire EDM and die-sinking EDM process parameters during the last few decades. Different articles are found to be related to the effectiveness of UV on different aspects of machining performance. This section of review article will analyse and gather data about the impact of UV on major parameters such as material removal rate (MRR), the surface roughness (SR) and tool wear rate (TWR).

### 5.1. Effect of UVA on Material Removal Rate

**Figure 41:**
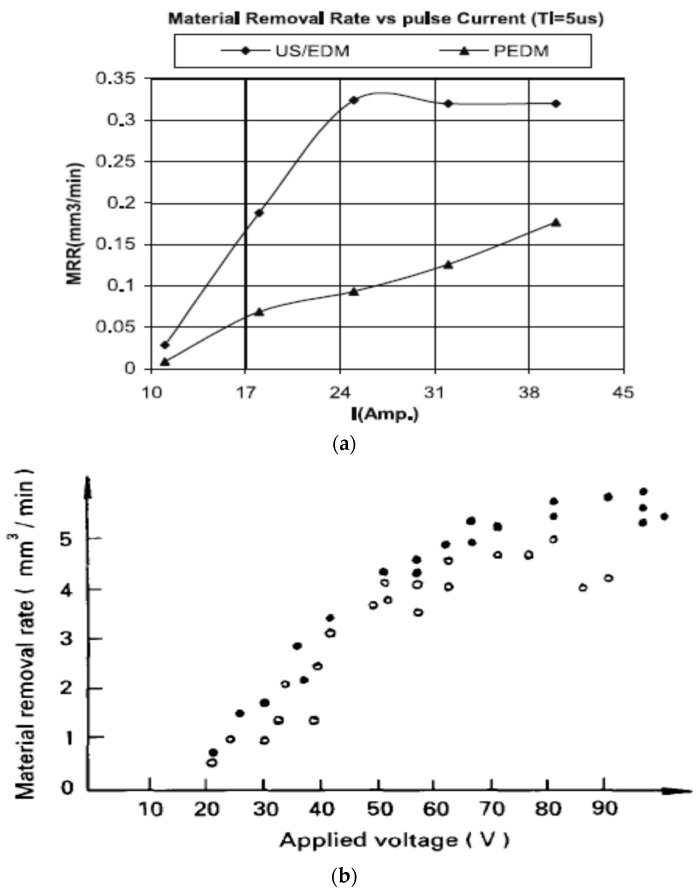
Effect of tool vibration on MRR versus discharge current (I) for US/EDM and pure EDM [105]; (Adapted from [105] with permission—© 2008 Springer). (**b**) Effect of Applied voltage on MRR ((hollow circle: 15 µm amplitude; full circles: 25 µm amplitude [12]. (Adapted from [12] with permission—© 1995 Elsevier).

MRR associated with ultrasonic vibration assisted EDM is always higher than that with pure EDM. Figure 41a shows MRR trend for both with and without UVA-EDM for pulse on time of 5 µs. As can be seen from the Figure 41, with the increase of discharge current, in general the MRR increases and for UVA-EDM the increase of MRR is relatively higher than conventional EDM. Also the increase of voltage enhances the MRR due to the increase of discharge energy (Figure 41b) [12]. Longitudinal vibration from tool electrode results in a compressive as well as rarefaction wave lead, other than micro-bubbles and ejecting stream. Forceful accelerated mass transfer across the spark gap is initiated by this wave front, which has similar functioning like a pump, thus causing a better evacuation of debris and introducing a new dielectric. Eventually, stable machining is achieved due to the reduction of short circuit as well as open circuit phenomena. Again, withdrawn of molten material from the pool as well as separation of the grains can be achieved by this compressive wave front, whereas rarefaction phase aids in creation of strong suction causing severe boiling of bulk materials as well as its removal via explosion [105]. Dielectric ionization and spark initiation are aided by the tool ultrasonic vibration, where half cycle of tool moving downward breaks down the dielectric molecules into smaller one and last half cycle of tool upward movement helps generating the bubbles due to the rarefication effect. In addition, acoustic–electric current is generated due to the transference of energy as well as momentum of ultrasonic vibration of the charged particles in the spark gap [55]. Collisional ionization induced by acoustic wave also aids in shortening the ignition delay. Compression and rarefication associated with the ultrasonic vibration also generate the cavitation phenomena. All these phenomena discussed above ultimately aids in higher MRR due to the application of UVA. Kremar at al reported 400% increase of MRR by synchronizing UVA frequency to the EDM frequency and improved evacuation of the debris [106]. Table 4 provides a summary of research studying the effect of UVA on the MRR during EDM.

### 5.2. Effect of UVA on Tool Wear Rate

**Figure 42:**
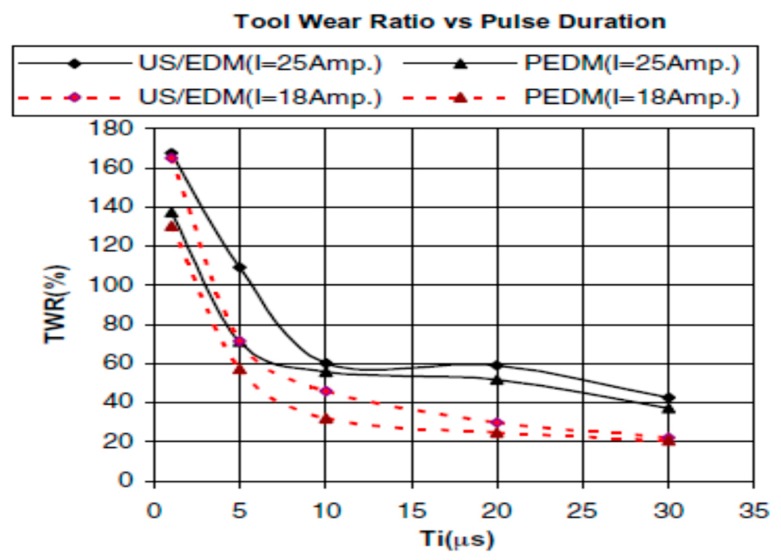
Effect of tool vibration on TWR versus pulse-on time (Ti) for US/EDM and pure EDM [105]. (Adapted from [105] with permission—© 2008 Springer).

As can be seen from Figure 42, UVA-EDM experiences higher tool wear ratio than pure EDM. Cavitation bubbles generated due to the UV application collapses near the surface of the tool and workpiece. Collapsing of these bubbles bring high velocity jet of dielectric liquid onto the surface thus resulting in a shock wave. Eventually this shock wave causes not only the pits generation on the tool surface but also creates the new-born heated surface which might be the reason for the higher tool erosion. The up-down movement of the tool causes the cavitation to take place mostly on the adjacent tool surface, therefore enhances the tool wear ratio with the addition of UVA [105]. However, Jiang et al. reported on the improved tool wear rate due to the improved gap status and weakened tool erosion [87]. A similar observation was reported by Tong et al. during ultrasonic vibration (workpiece) assisted micro-EDM. Their study reported on the proportional increase of discharge ratio with the increasing vibration frequency. This increase of discharge ratio aids in improved flushing and tool wear [20]. On the other hand, Ichikawa et al. reported improved tool wear due to the reduced lateral gap width between the tool and workpiece [102]. Srivasava also reported on improved tool wear ratio for cryo-cooled tool during UVA-EDM [56,57]. Table 5 provides some finding about tool wear ratio research during UV assisted EDM.

### 5.3. Effect of UVA on Surface Roughness & Metallurgical Characteristics

**Figure 43:**
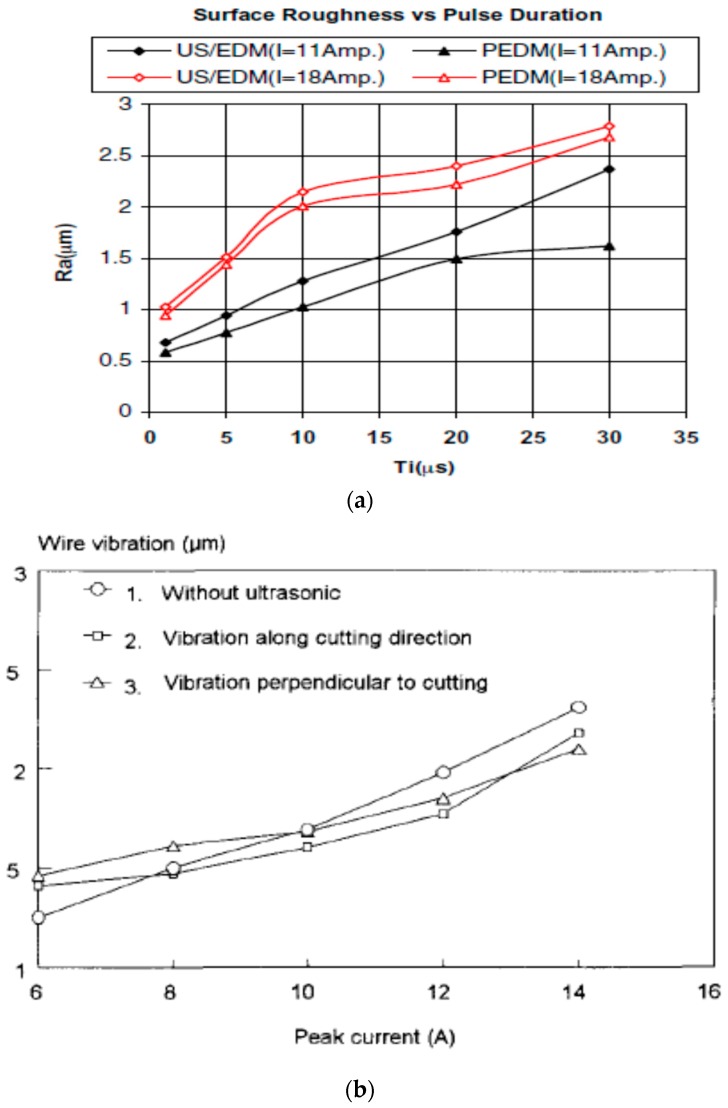
(**a**) Effect of tool vibration on surface roughness versus pulse-on time (Ti) for US/EDM and pure EDM [105]; (Adapted from [105] with permission—© 2008 Springer). (**b**) Effect of ultrasonic vibration on the machined surface roughness [72]. (Adapted from [72] with permission—© 1997 Elsevier).

UV assisted EDM process not only increases the MRR due to the application of UVA but also increases the roughness of the generated surface (Figure 43a). For this case, surface roughness of WC increases roughly 10 times of normal EDM. Roughness of generated surface increases both with the increase of pulse duration and pulse current. The reason behind this increment can be correlated with the reduction of ignition delay as well as larger average pulse energy. On top of that, higher drop of plasma pressure at the end of discharge associated with UVA-EDM causes large amount of melt to be ejected from the molten pool therefore generating the deeper craters [105]. With the increase of voltage, distance between tool and workpiece gets longer, causing the better flushing of debris. Eventually, higher cavitation occurs on both the tool and workpiece surfaces, thus enhances the surface roughness (Figure 43b) [45].

Nevertheless, Guo et al. reported on reduced surface roughness for UVA-WED (Figure 43b). The reason behind this might be the occurrence of multiple discharge points due to ultrasonic vibration, that reduces the average single discharge power, thus, reduces the surface roughness. Their study reported reduced tensile residual stress for similar reason [72]. Table 6 provides a summary of researches related to surface roughness obtained in UV assisted EDM.

## 6. UV Aided EDM for Hard to Machine Materials

The electro-discharge machining of ceramics became popular among the metal cutting industries because of its features and competitive cost. Lee et al. [50] studied the effects of ultrasonic vibration assisted EDM process on surface roughness and material removal rate during UVA-EDM of ceramics and found out that material removal rate for UV-A EDM is more compared to conventional EDM, while surface roughness is higher. Thoe et al. [54] used different tool materials like tungsten, silver steel and copper tool electrodes to drill the hole of 1 mm diameter. Ceramic nickel alloy was used as workpiece for UVA-EDM. Based on the experimental results, mild steel was considered as most elastic, while other materials failed, broke and had deformations. Use of suitable tool material is important in order to get effective UVA-EDM operation.

Titanium alloy (Ti–6Al–4V) is one of the hardest materials to cut using conventional machining processes. Ultrasonic vibration assisted EDM has been studied for machining this alloy [53]. Material removal rate for this technique was higher for UVA-EDM than without ultrasonic assistance (UA). A similar work was done by Wansheng et al. [62], as they applied ultrasonic vibrations into the micro-EDM process for drilling holes in Ti–6Al–4V. The experiment was performed to produce holes with diameter of 0.2 mm. UV assistance could improve the liquid flow and avoid the erosion of material. The machining stability and efficiency can be achieved by the improvement of pulse discharge ratio. In the investigation of Shabgard and Alenabi [124], the effect of copper tool vibration on the same material was analysed during UVA-EDM. Scanning electron microscope (SEM) was used for the analysis of surface integrity. By increasing the amount of normal discharges and decreasing the amount of arc discharges, enhancement in MRR was obtained. TWR and amount of cracks decreased when UVA-EDM was applied at the finishing stage. Due to a reduction of abnormal discharges, the process stability in UVA-EDM mode was much higher than in conventional EDM. Chen et al. [125] made an investigation by using kerosene and distilled water as dielectrics for UVA-EDM of Ti-6Al-4V. Comparison of results indicate that in kerosene dielectric, material removal rate is lower and tool wear rate is higher than in water. Tungsten carbide (WC) is a hard to cut material. However, this material is widely used in manufacturing, due to its physical and chemical characteristics, such as corrosion and wear resistance [6]. The surface integrity of WC (WC–10% Co) material could be considerably improved by implementing ultrasonic vibration to the tool during the EDM process [48]. Jahan et al. [23] reported an investigation where low-frequency vibrations were applied on the workpiece during micro EDM process for machining of WC. Goioganna et al. [126] used ultrasonic vibrations to decrease machining time and improve workpiece accuracy. In their experiment, a horizontal blind hole with an aspect ratio of 14 and diameter of 14 mm of the external circle was produced. Due to high aspect ratio and horizontal position of hole, some difficulties with debris removal were faced. Nevertheless, they achieved 58% reduction of machining time by using UVA -EDM and increased accuracy. In the experiment of Iwai et al. [58], machinability of polycrystalline diamond (PCD) was studied. Efficiency of EDM increased almost 3 times compared to conventional EDM with copper electrode. The experimental results demonstrate that the combination of EDM and ultrasonic vibration is an almost perfect method for machining of PCD material. The most popular area of PCD usage is in the cutting tool application due to its superior hardness, strong wear resistance and toughness. When pulse interval and width are constant, the processing speed directly depends on the peak current value. Ultrasonic vibrations has a unique chip ejection function during UVA-EDM but has a low impact on MRR [91].

Effect on machining properties of Si_3_N_4_ was investigated using EDM combined with ultrasonic vibrations [46]. It is reported that ultrasonic vibration must be applied after the transition time is over. It is also found that the large amplitudes do not always increase the MRR and the MRR of conventional EDM was 2 times lesser than that of UVA-EDM. The surface roughness also increases due to the ultrasonic vibrations. Impact of workpiece vibrations on FW4 welded metal characteristics during ultrasonic assisted EDM was studied by Shabgard et al. [89]. When short pulse on time was applied, material removal rate in UVA-EDM was 4 times greater than in conventional EDM. However, for long pulse on time, MRR is reduced by ultrasonic vibrations. In UVA-EDM with a short pulse on time, TWR was lower than the traditional EDM. The surface roughness in UVA-EDM was slightly larger than in traditional EDM. Shabgard et al. [88] investigated another similar work. Graphite was used as a tool electrode and the effect of UV combined with EDM on AISI H13 tool steel was studied. Inactive pulses were reduced by ultrasonic vibration of workpiece and the process stability was improved. They varied pulse duration and peak current parameters to observe their effect on MRR and TWR. The results show that, for low current and low pulse duration, MRR in UVA-EDM was 3 times higher than that of conventional EDM. The surface roughness was slightly better in UVA-EDM process. Through decreasing the short circuit pulses, average pulse energy was increased. Huang et al. [66] investigated the effect of UV assistance on EDM for manufacturing the micro holes on Nitinol (nickel and titanium alloy). The more the amplitude of ultrasonic vibration, the more effective is the process, with small electrode wear. High voltage leads to a high tool wear rate but a significant increase in MRR. The pre-sparking gap had same tendency for the TWR and machining efficiency. It was proved that ultrasonic vibration aided EDM is more effective compared to conventional EDM for machining difficult-to-cut materials. The summary for UVA-EDM of hard to cut materials is provided in Table 7.

## 7. Current Challenges & Future Research Scopes

There has been significant amount of works done on the feasibility studies of the UV assisted EDM using different dielectric mediums and applying vibration onto the workpiece, tool and dielectric. Many of those studies focused on investigating and comparing the performance of UV assisted EDM/micro-EDM with the traditional EDM/Micro-EDM. In addition, a significant amount of works has been carried out on studying the effect of both electrical and vibration parameters on the machining performance parameters such as MRR, tool wear and surface finish. However, a number of research questions remain unanswered, that should be considered by the future researchers as scopes for future research. Following are some suggestions for the future research on this area of EDM.

- One of the issues in UV assisted EDM or micro-EDM, is the accuracy of the parts or structures produced. This issue is more important to be considered in tool vibration assisted EDM, however, it was also found to cause inaccuracies in workpiece vibration assisted EDM. Due to the ultrasonic vibration, the spark gap changes continuously, which results in changes of overcut by the process. As a result, an irregularity in the machined hole diameters or difficulty in achieving sharp corners in the microstructures were observed. Future research should try to resolve those issues and investigate the ways of improving the part accuracy, while enhancing the MRR.
- Although there have been studies on investigating the surface topography and roughness generated in UV assisted EDM, in depth studies investigating the sub-surface microstructure and mechanical properties changes of the part machined by the process is still insufficient. In addition, for most of the cases UV assisted EDM generated higher surface roughness compared to traditional EDM process. Therefore, future research should focus on investigating the sub-surface microstructural changes after conventional EDM and UV assisted EDM to see if the application of ultrasonic vibration helped in minimizing the heat affected zone at the sub-surface and improve the microstructure at the sub-surface level. In addition, various mechanical properties including residual stress, tensile and fatigue properties of the UV assisted EDM process machined parts can be compared with the traditional EDM process machined parts.
- The current research on UV assisted EDM lacks from the sensing, monitoring and control of the process, which could possibly resolve the inaccuracy issue discussed in the previous point. Very few research studies have focused on this important issue. The in-situ monitoring of the sparking conditions at the spark and/or the width of the plasma channel and feedback to the control system to control either the electrode feed or vibration frequency/amplitude to maintain the stable plasma column width, will be able to improve the dimensional accuracy in UV assisted EDM/micro-EDM.
- The tool wear compensation during the UV assisted EDM can also improve the dimensional accuracy of the parts machined. There have been very few research studies on monitoring the tool wear and developing algorithms for tool wear compensation in UV assisted EDM, although there were many studies on the same for conventional EDM and/or micro-EDM. The authors believe that it is more important to monitor and compensate tool wear in UV assisted EDM to maintain the accuracy of the parts and structures.
- There have been very few studies on modelling the UV assisted EDM process in both liquid and dielectric mediums. Several studies have developed the models of MRR and SR based on the geometry of the crater sizes and Gaussian heat source without considering the ultrasonic vibration parameters, such as frequency, amplitude and vibration induced cavitation. It is important to note that unlike conventional EDM, the cavitation is a major physical phenomenon that should be considered during the modelling of the UV assisted EDM or micro-EDM. Therefore, future research should focus on more physics-based modelling considering the cavitation as secondary material removal phenomenon in addition to sparking induced craters. Moreover, the effect of vibration frequency and amplitude should be taken into consideration when modelling the UV assisted EDM or micro-EDM.
- In recent years, there have been a few studies that focused on integrating assistance of other physical phenomena with the ultrasonic vibration assisted EDM, such as UV assisted EDM with powder mixed dielectric, where more than one physical assistance was used. Future research studies should explore more on this area of hybrid machining by including the assistance of other physical principles with UV assisted EDM to further improve the machining performance of the conventional EDM process. Some examples can be combined magnetic and UV assisted EDM, UV assisted electro-chemical discharge machining (UV assisted ECDM), UV assisted EDM-ECM combined process, combined UV and laser assisted EDM and so on. A lot of research opportunities will open up with the development of new hybrid machining processes as suggested above, including initial feasibility study, performance characterization and optimization, modelling of the process and applications of the new process, to name a few.
- Finally, the application of UV assisted EDM and micro-EDM process could be broadened to semiconductor and micro and nanoelectromechanical systems (MEMS/NEMS) by investigating the machinability of new and innovative materials using the UV assisted EDM process. One of the major challenges in UV assisted EDM of semiconductor materials like silicon or germanium wafer is the recast layer formation and heat affected zone beneath the machined surface, that modify the electrical and thermal properties of the semiconductor materials. As UV assisted EDM/micro-EDM is found to minimize the recast layer thickness underneath the machined surface, UV assistance can be used with the wire EDM or micro-EDM to cut semiconductor wafers or machine 3D microstructures with semiconducting materials without making changes in important electro-thermal properties. Extensive research on this issue is needed before UV assisted EDM/micro-EDM be an acceptable machining process in the semiconductor industries.

## 8. Conclusions

Ultrasonic assisted EDM is a hybrid process that combines the physical phenomena of ultrasonic vibration with the EDM and/or micro-EDM process to improve the process stability and performance. The ultrasonic vibration was found to be applied effectively in both workpiece and tool electrode based on the applications and structures to be machined. This review paper provides an overview of the principle of EDM and ultrasonic vibration assisted EDM, as well as includes a comprehensive review of various aspects of ultrasonic vibration assisted EDM including major challenges and guidelines to resolve the challenges in the future research. It can be concluded from the literature that ultrasonic vibration assistance increases the MRR, surface roughness and tool wear ratio due to the reduced arcing, inactive pulses, cavitation effect and stable discharge. The MRR can be enhanced by increasing voltage, current and vibration amplitude. Increase of discharge voltage, current, pulse duration and ultrasonic vibration amplitude causes increased surface roughness, whereas higher frequency vibration results in a lower surface roughness due to the generation of multiple channel of discharge. On the other hand, TWR can be reduced by increased vibration amplitude, tool rotation and planetary affect. The ultrasonic vibration assisted EDM was found to be especially effective, where the flushing of debris becomes challenging, such as in deep micro-holes. The aspect ratio and inside surface quality of the micro-holes could be significantly improved by applying ultrasonic vibration in micro-EDM drilling of deep micro-holes. Ultrasonic vibration assisted EDM was also found to be effective during the machining of complex blind structures for multi-scale die and mould making. The ultrasonic vibration assisted EDM was also found to improve the surface integrity by reduction of the ineffective pulses, that is, arching and short-circuiting, on the surface, thus reducing the thickness of recast layer and residual stress. Finally, it can be concluded that ultrasonic vibration assisted EDM is an effective process to machine difficult-to-cut materials and complex multi-scale structures on a range of materials including metals and ceramics, thus, improving the process capability of the traditional EDM and micro-EDM process.

## Figures and Tables

**Table 1:** Summary of research conducted in gas UVA-EDM.

Process	Vibration Applied	Findings	References
Oxygen gas assisted EDM	Workpiece	Oxygen assisted EDM provides higher MRR, higher eroded particles size and also generates additional heat due to oxidation. Zero tool wear is possible for any pulse on time.	[24,25,26,27,28]
Air/oxygen gas assisted EDM	Workpiece	Oxygen offers higher MRR than air. Increase of voltage, current, amplitude, pulse on time but tool wall thickness increase MRR.	[29,30]
Air assisted EDM	Workpiece	UVA-EDM in gas increases MRR twice of gas assisted EDM however, it is still lower than conventional EDM.	[31]
Air assisted EDM	Tool	Material removal mechanism are associated with melting, evaporation, oxidation/decomposition, spalling, high pressure force, influence of UV.	[32]
Air/ oxygen gas assisted EDM	Workpiece	Modelling of single spark and removal mechanism of single spark is proposed.	[33]
Air/ oxygen gas assisted EDM	Workpiece	TWR is relatively less in gas UVA-EDM, also increase of amplitude increases MRR due to reduced short circuit and prevention of re-adhesion of molten materials on workpiece.	[34]
Air assisted EDM	Tool	Material removal for hard to machine materials is proposed to occur in four steps, formation of thermal stress, formation of micro-cracks, grain breakage, particles strips via thermal spalling.	[35]
Oxygen/argon/air assisted EDM	Tool & workpiece	MRR increases for addition of both CNT and vibration (long pulse duration). Altered material zone depth is reduced with combined effect of UVA-EDM and CNT addition to dielectric.	[36]

**Table 2:** Findings related to Ultrasonic Vibration applied on Tool.

Process Type	Process Description/Parameter	Remarks
EDM	First part of device consists of ultrasonic generator which is synchronized with pulse generator. Second part supplies high pressure dielectric, inducing cavitation affect.	Electrode tool form and proportions have an insignificant impact on sonotrode resonant frequency. FEM simulation illustrates that blade shape electrode has extra oscillations [39].
	Study about surface integrity of tungsten carbide (WC-10%) using EDX; SEM, microscopy.	SR and hardness in UAEDM are higher than EDM. Thickness of recast layer, heat-affect zone is decreased [48].
	Tool vibrated at frequency of 20 kHz. The gap between tool and workpiece periodically changes because of alternative pumping motion of front face of tool.	Machining stability increased by good evacuation of melted material, via vibrating the tool sinusoidally. MRR and SR increased by UAEDM as amplitude, voltage and discharge current increase [45].
	Investigation compares low and high frequency vibration of electrode, axial vibrating electrode and their combination on MRR, TWR, SR.	MRR increased to 35% in semi-finishing regime by vibro-rotary EDM. Combination of UVA and vibro-rotary EDM leads to enhanced TWR, MRR, SR [49].
	Study on optimization of flushing by vertical vibration of electrode and reduced electrode wear and process time.	Comparative TWR reduces by 21% and MRR increased to 11%. Low amplitude of vibration and high frequency enhance machining efficiency [51,52].
	Dielectric type, discharge peak current, pulse duration and abrasive size changed during experiment.	MRR in UA EDM higher than in pure EDM. Distilled water gives more MRR and less REWR than kerosene. SR in kerosene is better than distilled water in UAEDM [53].
	Ceramic was machined by UA EDM tool supplied with DC power supply.	MRR, SR increases as voltage of D.C source, discharge current and amplitude of tool rises [50].
	Effect of UVA on bubble behaviour during necking phenomena is investigated for smooth, convex and concave shape workpiece and tool surface.	Material removal from workpiece in case of convex is more productive [43].
	Ultrasonic vibration is applied on both tool and workpiece. Bubble formation characteristics is investigated using different amplitude and frequency.	Lifetime of bubble for UA applied on both electrodes is longer than conventional EDM before necking phenomenon. Expansion of bubble size directly proportional to Amplitude. Frequency rise increases the pressure decrease inside bubble [44].
	MRR, SR, EWR is measured by applying combined effect of magnetic force and UVA	Hybrid EDMUVAMF increased MRR, offers finer surface integrity. Peak current increases MRR; EWR and SR. Positive polarity has higher value of EWR than negative polarity [25].
	M2 grade steel machined by UVA cryogenically cooled copper electrode.	Surface integrity and out of roundness is better in UAV-EDM. High frequency pumping motion increase MRR [56].
	Three types of vibration methods are used: axial, flexural, complex	3 times higher Removal rate achieved in axial, complex vibration. Surface roughness is almost the same for all vibration [58].
	Effect of UV, abrasive, electrode polarities, amplitude and conductive layer are investigated for ceramics.	Negative polarity has better machining effect. Good EWR is achieved by positive polarity. MRR increases twice compared to EDM. Surface roughness enhanced after Ultrasonic vibration application [46].
	UVA pulse EDM is used for hole producing.	Voltage rise increases MRR. MRR is directly proportional to the amplitude of vibration [12].
	Electrically non-conductive ceramic coated nickel alloy is investigated.	The need for Feedback of force control system was explored. MRR is reported more in UVA [54].
	Transient recorder recorded pulse train results received from UVA-EDM.	Ultrasonic vibration significantly decreases inactive pulses [55].
	Simulation studies of debris removal is conducted	With the increase of amplitude and frequency, fluid circulation enhances which aids in debris removal. Even with UV application higher aspect ratio hole above 4 is difficult to fabricate [82].
Micro-EDM	Electrode vibration with inclined feeding of electrode was investigated.	Drilling depth increases when 15 degree of upward inclining feeding is applied. Downward feeding and 10 degree inclining leads to best performance. Compared to conventional horizontal EDM, the depth increased by 75% [71].
	Microelectrode array is fabricated by reverse UVAEDM.	Machining time at different voltage values was less for UV assistance. Flushing of debris increases thus leads to good surface quality [63].
	4 axis EDM tool with UV assistance is developed. Diameter of <0.2 mm and ratio depth to diameter of 15 drilled.	Combination of UA and rotating of single-notch electrode is beneficial for small and deep hole machining in titanium alloy. Machining stability and surface quality gets better [62].
	Authors introduces method of micro-EDM process monitoring, by counting discharge pulses.	For appropriate calculation of the depth of cut ultrahigh-speed electronic circuit was developed. By prognosis method, the relationship between discharge pulse, number, energy and machining types is explained [64].
	UVA is used for decreasing secondary discharge and the wear of electrode. The effect of feeding depth is observed.	Hole tapering become less and deeper hole (1000 µm) is possible. With UVA combination d < 0.1 µm achieved [65].
	High aspect ratio micro features were machined by Reverse Micro EDM. Investigation focused on debris motion and magneto restrictive device provide vibration frequency.	In Reverse Micro EDM process debris size allocation is asymmetrical and size lies between 90–950 nm range. Upward movement of plate leads to flush out of debris from gap. Downward motion leads to reverse direction flow [70].
	Investigation about direct and indirect application of UV on EDM.	Application of direct UV increases process speed of micro EDM by 40%.Low frequency vibration eliminates deviation in accuracy of shape. [69]
	Article describe new method of machining, actuation of Micro-EDM electrode in X, Y directions within ±100 µm. (Orbital trajectory)	Better surface finish and reduced EW is achieved. Orbital trajectory improves bottom quality of blind hole. [68]
	UVA used in machining of Nitinol	High amplitude of vibration causes small electrode wear and also has positive effect on efficiency. High voltage increases electrode wear but also increases efficiency [66].
Wire-EDM	Scheme of UVA-WEDM is presented	Roughness and residual tensile stress are reduced and 30% increases of cutting efficiency occurs. Highest cutting effectiveness achieved, when orientation was in same direction with cutting direction [72].
	Vibration was applied on workpiece and electrode. Comparison work is performed using different frequencies on both methods.	Workpiece vibration was better than electrode vibration for debris circulation. Cutting rate is enhanced by 1.5 times and 2.5 times for workpiece vibration compared to electrode vibration and pure EDM respectively. SR is better than conventional EDM, in both methods [76]
	Feed rate, frequency and gap voltage of WEDM are taken as input parameter and MRR, Kerf width as output parameter.	MRR, Kerf width values depend on capacitance value. Frequency of vibration contributes 10.88% increases of the MRR. Low frequency enhances machining performance of Inconel 718 [83].
	Authors developed mechanism and simulation method to investigate the effects of UVA on WEDM.	Cutting rate, Surface roughness, Discharge point (shifted), Rupture on wire (probability decreased) were improved [73].
	Accurate spindle with flexibility and high resistance to corrosion is designed.	Greater MRR obtained via UVA. Which has high impact during roughing condition [78].

**Table 3:** An overview of researches related to Ultrasonic Vibration application on Workpiece.

Process Type	Process Description/Parameter	Remark/Results
EDM	Stainless steel is machined by applying Low frequency, amplitude vibration to workpiece.	MRR achieved by UAV was higher than Pure EDM. The maximum result obtained by 600 Hz, 0.75 µm. MRR increased up to 23%. TWR, SR in pure EDM was bigger (worst) than UVA-EDM [85]
	MATLAB Fuzzy-Toolbox is used as simulation application.	Fuzzy LC reduced the rate of accretion formation from 27% to 14.6%. It is approved that FLC is better than PID controller [86]
	Voice Coil motor is used in UAVEDM for small hole performance.	TWR for ED M is 22%, whereas for UVA-EDM it is 8% and 7% in motor coil vibration. Machining efficiency become 5 times higher for UVA-EDM. Perforation time gradually decreased when f = 1000 Hz and current rises from 0.2 A to 1 A [87]
	Optimum machining parameters are determined by applying UV on workpiece during machining AISIH13 tool steel.	The small pulses, low current lead to 3 times more MRR compared to PEDM. Surface roughness also was higher in UVA- EDM. Short circuit pulses, arcing are reduced and flushing becomes better by UVA [88]
	Performance parameter of FW4 welded metal machined by UVA-EDM.	MRR was 4 times higher and TWR was reduced for short pulse on-times. In long pulses. Surface roughness was slightly larger in UVA-EDM [89]
	AISI 1045 steel processed in Gas medium in UA EDM.	MRR in gas medium was 5 mm^3^/min for EDM, whereas in UVAEDM it was 9 mm^3^/min. Discharge current, pulse duration has the most strong impact on UEDM [31]
	Rotary magnetic field assisted dry EDM and Tool with 2 eccentric hole, brass tool were investigated.	MRR of Brass tool is larger than the copper tool. Brass tool has a larger SR, EWR than copper. tool with two eccentric holes lead to higher MRR and lower SR. Magnetic field could drive out the debris from gap and leads to increased EWR [90]
	Combination of UVA- EDM and polycrystalline diamond is explored and bronze-bonded diamond grinding wheel was used.	The increase of the peak current also rises processing speed. Open circuit voltage of 160 V induces high processing speed [91]
	Horizontal UVA-EDM is simulated by Finite Element Methods.	Surface roughness improved from 2.543 to 2.050 by HEDM.MRR in H-EDM increased 3 times, from 0.49 mm^2^/s to 1.77 mm^2^/s [92]
Micro-EDM	The micro- vibration of workpiece is performed by application of high-frequency sine-wave voltage, to manage piezoelectric actuator.	Machining stability improved. Machining efficiency increased by 18 times, when f = 6 kHz, A = 3 µm [20]
	VA servo scanning 3D M-EDM is machined via tool ES layer-by-layer according NC code. Vibration fulfilled adopting a piezoelectric (PZT) actuator	Regular discharge ratio and stability of machining is improved. Machining efficiency is directly proportional to values of frequency and at specific parameter, it is 6.5 times higher than machining without vibration. Dwell time is increased by LCV (lower scanning velocity) [93]
	Piezoelectric self-adaptive micro-EDM, which could make self-regulation is investigated.	Condition for less TWR and improved stability is provided [94]
	Simply analytical model of low frequency workpiece vibration is presented in deep hole drilling process. Experiment is performed in different capacitance and gap voltage.	Low frequency micro-EDM productivity depends on K(v) parameter, which is the ratio of Max vibration acceleration to gravitational acceleration in same direction. In K(v) > 1 the performance increase as frequency of vibration increase. However, accuracy and surface quality decrease in certain maximum point of frequency [23]
	Current, voltage waveforms, electrode feeding parameters are recorded and analysed.	Reduced machining time, arcing event and process time are reported [22]
	EDM with Ultrasonic vibration of workpiece is presented in paper.	For copper material MRR was almost 1.2 mm^3^/min and 0.4 mm^3^/min for UVAEDM and EDM respectively. MRR for steel is 0.22 mm^3^/min(UVAEDM), whereas for TEDM MRR = 0.045 mm^3^/min. Efficiency of UAEDM 8 times greater than TEMD when workpiece thickness was 0.5 mm [96].
	Optimal parameter are defined by ANOVA and Signal to Noise ratio.	Based on ANOVA result, parameter condition obtained for best MRR, are at 60% of peak power vibration and 3300 PF capacitance.Experimental and theoretical results match at 95% [98]
	Application of UV on workpiece using Powder mixed dielectric fluid is investigated.	Machining time for graphite powder was significantly less and reached to a minimum value. Compared to pure EDM, surface machined by this powder was better (well defined craters) 15 g/L is the best concentration of powder for high surface quality [97]
	Application of UVA in fluid is performed by probe-type vibrator in ceramic machining.	Dielectric vibration enhanced MRR, machining depth, surface topology, stability of process. UVA without carbon nanofibers is ineffective. Maximum depth of hole achieved through 10 µm amplitude vibration [100]
	An effect of UVA dielectric liquid for obtaining best machining parameters is achieved by Taguchi method via usage of micro-MoS_2_ suspension in fluid.	MRR and Surface quality increased after addition of micro-MoS_2_.Presence of MoS_2_ has significant influence on particle.Cu is better machined rather than Cu-W, Ag-W with MoS_2_ powder [21]
	Reaction bonded silicon carbide is machined due to the carbon nanofibres supplementation into dielectric liquid with help of ultrasonic cavitation assisted EDM.	Addition of carbon nanofibres into dielectric liquid decrease deposition of the tool on surface of workpiece. For higher amplitude, lower rate of deposition is received. By decreasing the distance between workpiece and oscillator, prevention of tool material deposition is achieved. High accuracy is obtained through inclined workpiece and appropriate finishing time [101]
	EDM process is carried out by vibrating worktable,	Noncircular micro-electrode was successfully machined by UVA-EDM with diameter (<200 µm). Spark number increased significantly, hence machining efficiency enhanced [95]
	Machining process with Ultra-small discharge energy fulfilled by UV application on dielectric liquid is presented	Width of lateral gap, machining time and TWR become smaller. Vibration amplitude had not got big effect on characteristics of machining [102]

**Table 4:** Findings related to MRR performance of UV assisted EDM.

Process	Vibration Applied	Findings
EDM	Electrode	MRR improved significantly due to minimised arcing, reduced inactive pulses, open circuit. Machining rate reduces with the increase of depth due to the chip accumulation [107]. UVA force field [108], stirring in fluid, cavitation and micro-streaming induces improved flushing [109,110]
	Electrode	Increase of MRR (10–400%) is due to the enhanced pumping of dielectric [40,106]
	Electrode	Combined vibro-rotary enhances 35% MRR compared to vibro-EDM [49]
	Electrode	Combined UVA-EDM provides threefold MRR than USM [111]
	Electrode	UVA-EDM improves MRR by 49% due to the high pulse frequency. Rotary ultrasonic EDM reduces MRR due to its reduced machining stability [80]
	Tool & workpiece	Higher MRR is reported using vibration to both electrode compared to non-vibration as well as vibration to one electrode due to improved flushing [44]
	Workpiece	MRR for planetary and UVA-EDM appears to be as high as 6539 µmm^3^/s. 29 Aspect ratio micro hole can be fabricated using combined effect [112]
	Workpiece	MRR increases with the capacitance and voltage but decreases with amplitude. T = 150 µs, A = 4.919 µm and C = 0.01 µF are found to be optimal [113]
	Workpiece	UVA-EDM has eight times higher efficiency than normal EDM and it also offers higher aspect ratio hole [96]
	Tool	MRR increases with the increment of voltage, current and vibration amplitude [45]
	Tool	Higher MRR achieved due to synchronization of EDM frequency with UV frequency [39]
	Tool	Addition of UV and CNT separately increases MRR. For longer pulses CNT addition can enhance MRR for UVA-EDM due to the reduced abnormal discharges and increase of spark gap [36]
	Tool	47% enhancement in MRR was reported for 30% increase of vibration power [114]
	Tool	Increased MRR due to stable discharge and reduced arcing are caused by UVA [54]
	Tool	Combined micro-EDM and micro USM enhances MRR [115]
	Tool	The rise in amplitude rate of UVA causes increase in MRR [34]
WEDM	Wire	Both vibration parallel and perpendicular to cutting direction enhances MRR. Higher frequency vibration results in higher MRR due to multiple channel of discharge generation [72,73]
	Wire	25–37% enhancement of erosion capacity is reported [116]
Die-sinking EDM	Tool	UVA can enhance MRR up to fourfold at low pulse on time and current [105]
	Tool	MRR for cermet tool tip is higher than Cu tool tip [117]

**Table 5:** Findings related to TW performance of UV assisted EDM.

Process	Vibration Applied	Findings
EDM	Electrode	Tool wear increases due to better sparking frequency caused by UVA [110]
	Electrode	Higher tool wear was reported due to the porous nature of the tool [40]
	Electrode	UVA-EDM enhances TWR significantly whereas addition of CNT reduces TWR for shorter pulse on time [36]
	Axial vibration	Tool wear is mostly affected by increase of amplitude followed by increase of tool rotation due to enhance spark efficiency as well as better flushing [49]
	Electrode	Rotary EDM reduces tool wear comparatively than UVA-EDM [80]
	Electrode	UVA-EDM with cryogenically cooled tool experience less tool wear due to the formation of smaller crater size and increase of recast layer [56,57]
	Electrode	Tool wear can be reduced up to 18% by increasing power of vibration [114]
	Electrode	TWR in gas medium was reasonably low [34]
	Workpiece	Tool wear is much more enhanced with combined vibration and planetary effect than individual one [112]
	Workpiece	Less tool wear reported for UVA-EDM than pure EDM [20]
	Workpiece	TWR is reported to be reduced from 23% to 6% due to enhanced gap condition as well as less tool erosion [87]
	Dielectric	Reduced short circuit and abnormal discharge caused by UVA-EDM results in reduced tool wear [102]
WEDM	Wire	Higher frequency also aids in reduced wire breakage due to the shifting of discharge point [72,73]
Die-sinking EDM	Tool	Tool wear ratio is higher for UVA-EDM due to tool oscillation and cavitation effect on tool surface [105]
	Tool	Cermet tooltip experiences lesser TWR due to the presence of TiC (more resistance) than copper tool tip [117]

**Table 6:** Findings related to Surface characters performance of UV assisted EDM.

Process	Vibration Applied	Findings
EDM	Electrode	Improved roughness is reported due to the lateral movement of molt and forced flushing [118]. Debris generated in UVA-EDM appeared to be globular solid in shape compared to those without UVA [110,119]
	Electrode	Vibration assistance improves surface roughness and reduce white layer thickness formed. The surface microhardness and residual stress are not affected significantly [40,106]
	Axial vibration	Roughness increases for UVA-rotary EDM compared to pure EDM and rotary EDM [49]
	Electrode	Surface integrity is not much affected by UVA [111]
	Tool	Roughness increases with the increment of voltage, current and vibration amplitude [45]
	Tool	Good homogeneous distribution of wreck layers had positive affect on brightness and roughness of surface [120]
	Tool	MRR increased from 0.49 mm^3^/s to 1.71 mm^3^/s with the aid of horizontal UVA-EDM [92]
	Tool	CNT addition in general reduces roughness and cracks compared to pure EDM. UAV-EDM increases the roughness whereas addition of CNT to UVA-EDM can reduce the roughness [36]
	Tool	The amount of resolidified material is less in UVA-EDM, because of better circulation of fluid at electrode gap [121]
	Workpiece	Roughness increases with the increase of pulse duration, voltage and current. Pulse duration is the most influential parameter and voltage is the least [122]
	Workpiece	Discontinuous vibration creates smaller cracks due to the reduced resolidifcation of molten materials in each crater [123]
WEDM	Wire	Crater shapes changes from rounder shape (WEDM) to elliptical shape (UVA-WEDM) due to the shifting of discharge channel [72,73]
Die-sinking EDM	Tool	10% higher roughness is reported for UVA-EDM due to shorter ignition delay time and higher average pulse energy [105]
	Tool	Cermet tool tip offers better roughness than Cu tool tip [117]

**Table 7:** Summary of hard to cut material machined by UVA-EDM.

Material	Process	Findings	Reference
Ti alloy (Ti-6Al-4V)	UVA-EJM (Electrochemical Jet Machining is a localized form of ECM where jet of electrolyte via nozzle(cathode) is used as a tool.	UVA improved aspect ratio of the grooves, reduced formation of passivation layer by 23 % and reduced Ra by 31 %.	[127,128,129]
Cobalt-Chrome (Co-Cr)	UVA Micro-EDM	Greater values of vibration will lead to increase in surface roughness and transversal dimension of craters. Smaller craters observed are produced by ultrasonically induced cavitation. This may lead to significant increase in MRR.	[130]
Tungsten Carbide (WC)	UVA Micro-EDM	Increase of MRR as well as surface quality and decrease of EWR were observed. The optimal vibrations are found to be of 750 Hz frequency and 1.5 µm amplitude.	[131,132]
High-Speed Steel	UVA WED turning	UVA aids in increased MRR per discharge, reduced tensile and thermal stresses. High rotational speed and low power result in better surface quality.	[25,133]
Stainless steel 304	UVA-EDM	Application of low-frequency vibrations leads to higher MRR (about 23% increment), especially for vibrations of 600 Hz frequency and 0.75 µm of amplitude. Also tool wear rate and surface roughness were found to be lower.	[85,112,134]
Steel 45	UVA-EDM	Collapse of cavitation due to UVA leads to shock wave which generates erosion effect on the surface of the workpiece.	[135]
